# The dynamic relationship between hearing loss, quality of life, socioeconomic position and depression and the impact of hearing aids: answers from the English Longitudinal Study of Ageing (ELSA)

**DOI:** 10.1007/s00127-021-02155-0

**Published:** 2021-08-12

**Authors:** Dialechti Tsimpida, Evangelos Kontopantelis, Darren M. Ashcroft, Maria Panagioti

**Affiliations:** 1 Faculty of Biology, Medicine and Health, School of Medical Sciences, Institute for Health Policy and Organisation (IHPO), The University of Manchester, Oxford Road, Manchester, M13 9PL UK; 2 Faculty of Biology, Medicine and Health, School of Health Sciences, Institute for Health Policy and Organisation (IHPO), The University of Manchester, Manchester, UK; 3 Faculty of Biology, Medicine and Health, School of Health Sciences, NIHR Greater Manchester Patient Safety Translational Research Centre, The University of Manchester, Manchester, UK

**Keywords:** Hearing loss, Psychosocial wellbeing, Depression, Hearing aids, Ageing, Social epidemiology

## Abstract

**Purpose:**

The adverse impact of hearing loss (HL) extends beyond auditory impairment and may affect the individuals' psychosocial wellbeing. We aimed to examine whether there exists a causal psychosocial pathway between HL and depression in later life, via socioeconomic factors and quality of life, and whether hearing aids usage alleviates depressive symptoms over time.

**Methods:**

We examined the longitudinal relationship between HL and depressive symptoms (CES-D) applying dynamic cross-lagged mediation path models. We used the full dataset of participants aged 50–89 years (74,908 person-years), from all eight Waves of the English Longitudinal Study of Ageing (ELSA). Their quality of life (CASP-19) and their wealth were examined as the mediator and moderator of this relationship, respectively. Subgroup analyses investigated differences among those with hearing aids within different models of subjectively and objectively identified HL. All models were adjusted for age, sex, retirement status and social engagement.

**Results:**

Socioeconomic position (SEP) influenced the strength of the relationship between HL and depression, which was stronger in the lowest versus the highest wealth quintiles. The use of hearing aids was beneficial for alleviating depressive symptoms. Those in the lowest wealth quintiles experienced a lower risk for depression after the use of hearing aids compared to those in the highest wealth quintiles.

**Conclusion:**

HL poses a substantial risk for depressive symptoms in older adults, especially those who experience socioeconomic inequalities. The early detection of HL and provision of hearing aids may not only promote better-hearing health but could also enhance the psychosocial wellbeing of older adults, particularly those in a lower SEP.

**Supplementary Information:**

The online version contains supplementary material available at 10.1007/s00127-021-02155-0.

## Introduction

According to global estimates, over 1.3 billion people live today with some degree of hearing loss(HL) [1], and one-third of people above 65 years old are affected by disabling HL [2]. HL is the leading cause of morbidity among older adults in England [3, 4]. However, it is a highly underdiagnosed [5] and untreated chronic health condition [6, 7], which is quite alarming, as HL’s adverse impact extends beyond auditory impairment and may affect the individuals' mental wellbeing and their full participation in society [2, 8]

A recent systematic review and meta-analysis [9] found that HL was associated with 1.47 higher odds of depression in older adults (95%CI 1.31–1.65). However, the evidence had large inconsistency and the studies included had substantial heterogeneity (*I*^2^ = 83.26%). To date firm evidence about the psychosocial mechanisms implicated in the relationship between HL and depression is lacking [10], especially in a longitudinal context. In addition, despite the fact that studies consistently show that HL has a tremendous impact on quality of life [11–13], little is understood about the specific mechanisms involved in the relationship between HL and the multidimensional concept of quality of life [11, 13–15].

Moreover, evidence suggests that the use of hearing aids might moderate the relationship between HL and depression by alleviating depressive symptoms in older people with HL [10, 16, 17]. The Cochrane review of Ferguson et al. [18] had concluded, however, that the quality of evidence for the impact of hearing aids on the adverse effects of HL is very low; thus, large prospective studies are needed to raise the current quality of evidence [19] and further explore the potential moderating effect of hearing aids on the association between HL and depression. Given the rising prevalence of HL potentially due to social and lifestyle factors [2, 20], it is crucial to fill the above knowledge gaps, as depression and HL are responsible for enormous public health costs, morbidity and mortality [21].

This study aims, therefore, to (a) assess whether there exists a causal psychosocial pathway between HL and depression in later life, via socioeconomic position (SEP) (moderator effect) and quality of life (mediation effect), and (b) to investigate the moderating effect of hearing aids in reducing the risk of depression among older adults with HL.

## Method

### Study population

The analyses used the full dataset from the English Longitudinal Study of Ageing (ELSA). The ELSA is a longitudinal prospective cohort study that collects multidisciplinary data from a representative sample of adults aged 50 years and above in England [22]. The study has an ongoing 2-year follow-up longitudinal design with repeated measures of core variables over numerous Waves. Thus, it allows for an exploration of change in hearing acuity levels and trajectories on the social, wellbeing and economic impacts of such a change.

The ELSA follows the sampling strategy of the Health Survey for England (HSE), which ensures that every address on the small users’ Postcode Address File (PAF) in England has an equal chance of inclusion. Field household contact rates of over 96% were achieved. The study excluded cases not belonging to the target population through ‘terminating events’, such as deaths, institutional moves and moves out of England since taking part in the HSE [23].

As the ELSA follows a longitudinal design, the sample is comprised of a sequence of observation on the same individuals across Waves and the refreshment samples (Cohorts 3, 4, 6 and 7) [24]. Our analyses used the full dataset of participants aged 50–89 years, from all eight Waves of the ELSA, spanning 2002/3 to 2016/7 (74,908 person-years). We present a summary of the exact number of interviews by each Wave, and the fieldwork period as Supplementary Material 1 (page 1, item 1) [25].

We further analysed a sample of 8529 adults aged 50–89 years from Wave 7 that had an assessment in their hearing by both self-reported measures and who consented to assessment by a qualified nurse via a hearing screening device [26], and who did not have an ear infection or a cochlear implant.

All participants gave written informed consent at the recruitment Wave to participate in the ELSA and at each subsequent Wave. Ethical approval was granted by the National Research and Ethics Committee [25]. The authors assert that all procedures contributing to this work comply with the relevant national and institutional committees' ethical standards on human experimentation and with the Helsinki Declaration of 1975, as revised in 2008.

### Outcome measures

#### Depression

An eight-item short version of the Centre for Epidemiologic Studies Depression (CES-D) Scale was administered in the ELSA to assess clinically significant symptoms of depression [27]. The respondents had to indicate their feelings much of the time over the week before the interview, by confirming or not the particular feeling, respectively [27]. The questions and the eight-item short version CES-D scoring criteria are presented in the Supplementary Material 1 (page 1, items 2 and 3).

### Exposure measures

#### Hearing loss

The ELSA uses a self-reported measure of hearing in each Wave [28]. The category of self-reported HL consists of a merged category of those that rated their hearing as fair or poor on a five-point Likert scale (excellent, very good, good, fair or poor) or responded positively in the question about whether they find it difficult to follow a conversation if there is background noise (such as TV, radio or children playing).

Objective measurement of hearing acuity via HearCheck™ Screener was available only in the ELSA Wave 7. Hearing acuity was classified by the hearing performance in HearCheck™ Screener and defined as > 35 dB HL at 3.0 kHz in the better-hearing ear, a level where intervention has been shown as definitely beneficial [6].

We carried out additional work in a separate study [5] to examine self-reported data validity. We found that the self-reported hearing measure correctly classified seven in every ten people with objectively assessed HL via HearCheck™ Screener. In that work, we also proposed an improved categorisation of self-reported hearing difficulties. However, since additional variables of self-reported data were not available in Waves other than Wave 7 to allow for the use of these improved categories of self-reported data, we assumed for the scope of our analyses the available self-reported measure as a suitable indicator of HL.

### Mediator

#### Quality of life

The CASP-19 Scale is the measure of the quality of life used in the ELSA [29]. The measure uses 19 items, covering four domains: four items for control (C), five items for autonomy (A), five items for self-realisation (S) and five items for pleasure (P). The questions of all domains and the scoring criteria are listed as Supplementary Material 1 (page 1, item 4).

### Moderators

#### Socioeconomic position

Educational level was used as an exogenous socioeconomic variable, whose value is independent of other variables' states in the models. It was modelled in five educational level categories as presented in the ELSA datasets: degree/higher education; A level (Level 3 of the National Qualifications Framework); O levels CSE (Certificate of Secondary Education); foreign/other; no qualifications. We considered wealth as the most appropriate SEP indicator due to the sample's age (aged 50 years old and above) because the wealth status captures the SEP in both the later stages of active professional life and retirement period [30]. Wealth was examined as a moderating (intermediate) variable of depression. The variable is provided in the ELSA dataset in quintiles of the net total non-pension wealth, as reported at the household unit level (first quintile highest and fifth quintile lowest) [28]. The grouping of wealth in the ELSA into quintiles reduces the measurement error and allows the comparison of the health measures across the equally sized groups within the given population [31]. Thus, those in the highest wealth quintile refer to those in the top 20 %, while those in the lowest wealth quintile refer to those in the bottom 20 % in each ELSA Wave. The full definition of the net total non-pension wealth and the cut-off points for the wealth groups are presented in the Supplementary Material 1 (page 2, items 6 and 7).

#### Hearing aid use

As regards to the questions covering treatment for HL, the participants in Wave 7 were asked whether they ever wear hearing aids with potential answers being: (a) ‘Yes, most of the time’, (b) ‘Yes, some of the time’ and (c) ‘No’.

#### Covariates

We controlled for age, which influences the associations between HL and depression [32]. Age was entered in all structural equation models (SEMs) as a continuous variable, to maximise power. We considered sex as a covariate, as previous research has shown that there are differences in HL prevalence among men and women [7]. We also considered retirement status, and social engagement as covariates; the retirement status may confound the associations, and the degree of social engagement has been proposed to explain the association between HL and reduced mental wellbeing in older adults [15, 33, 34]. Retirement status was dichotomised to those who were retired or not, according to the self-reported employment status. A continuous measure of social engagement was included in the analysis, derived from a set of eight binary variables, asking whether the respondent is a member of various civic and social organisations. The eight binary variables are in the Supplementary Material (page 2, item 8).

### Data analysis

We fitted dynamic cross-lagged path models (CLPMs) to estimate HL and depression's association over time. CLPM is a type of structural equation model used where two or more variables are measured at two or more occasions (repeated measures), and the focus is on the associations (often causal theories) with each other over time. In the path analysis part of the generalised structural equation models (GSEM), we used the full dataset from the eight Waves (74,908 person-years), to strengthen the causal argument between HL and depression over time. Minimum Akaike's and Schwarz's Bayesian information criteria (AIC) and (BIC) values informed on the best-fitting recursive path models. Following these criteria, we considered HL an exogenous predictor with a uni-directional effect on wealth, which worked as an endogenous outcome variable in the models. Additionally, we examined wealth as a moderating/intermediate dependent variable of depression, which was the outcome variable in the dynamic CLPMs.

The concept of quality of life functioned as an endogenous mediator variable that intervenes between HL and wealth, explaining the relation between HL and SEP [35]. We represented the concept of quality of life using the confirmatory factor analysis (CFA) approach to generate a latent variable in each Wave. We calculated a standardised factor score that weights each item by their salience (loadings and correlation with the other items), rather than their mean or summative scores to allow each item to have its own variance. In the CFA models, we used the alpha reliability estimates to estimate reliability.

Exponentiated coefficients and summary statistics for each Wave are reported. Also, mixed-effects regression was used to estimate the repeated measures' interdependence on the same participants using the intraclass correlation coefficient (ICC) and the variance across the repeated measures. We applied Sobel's test to calculate the significance of mediation in the CLPMs. Finally, we calculated the percentage of the total effect that is mediated (indirect effect/ total effect) as the measure of the extent of mediation in each CLPM.

Regarding missing data, we used a full information approach that utilised available information in the presence of missing values on one or more variables, without the use of listwise deletion, applying the *method (mlmv)* command in Stata. That method provides a maximum likelihood estimate using all observed values, assuming joint normality and that the missing values were missing at random. As we conducted analyses combining data from multiple Waves, we applied the longitudinal weighting using *svy* commands, to account for any bias arising from Wave non-response and attrition (for reasons of death, illness or lack of interest) [24]. The two-tailed significance level was set at ≤ 0.05. All data were analysed using Stata version 14 [36].

As a sensitivity analysis, we conducted subgroup analyses of the path models to explore the use of hearing aids as a moderator variable and investigate potential differences in the structural relationships among those who reported use of hearing aids some of the time or most of the time, respectively, compared to those that did not use hearing aids. As a second sensitivity analysis, we fitted similar models to investigate potential differences in parameter estimates of depression in Wave 8 of the participants, according to the (a) self-reported measures of hearing difficulties, (b) the improved categories of self-reported data [5], and (c) the objective hearing measures via HearCheck™.

## Results

### Outcome measures

The participants’ non-modifiable demographic factors, and prevalence of HL and elevated depressive symptoms in eight Waves of the ELSA are provided in Table 1 of the supplementary material.

**Fig. 1:**
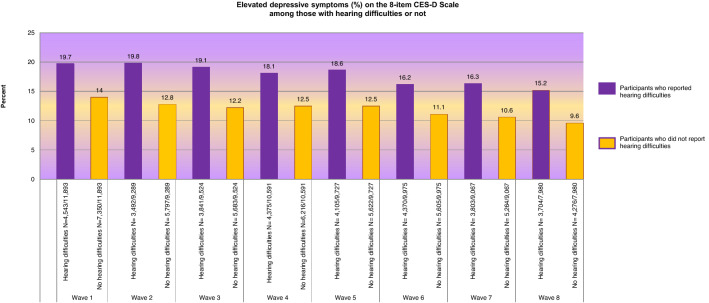
Clustered stacked bars for the probability (%) of elevated depressive symptoms a (on the eight-item CES-D Scale) among those with self-reported HL b or not in the eight Waves of the English Longitudinal Study of Ageing (ELSA). **a** Elevated depressive symptoms: the cut point of the eight-item dichotomous response scale (greater than or equal to four symptoms on the Scale) (8CES-D⩾4). **b** Self-reported HL (hearing difficulties): the sum of those that rated their hearing as fair or poor on a five-point Likert scale (excellent, very good, good, fair, or poor), or responded positively in the question whether they find it difficult to follow a conversation if there is background noise (such as TV, radio or children playing)

Figure 1 shows the probability (%) of elevated depressive symptoms in eight Waves of the ELSA. The relative risk for depressive symptoms was higher for those who had reported HL than those who had not reported HL (ranging from 1.40 in Wave 1 to 1.58 in Wave 8).

**Fig. 2:**
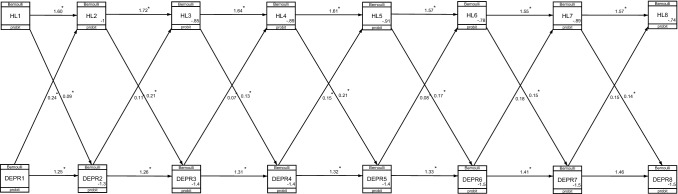
Cross-lagged path diagram model for hearing loss (HL) and depression (DEPR) in the eight Waves of English Longitudinal Study of Ageing (ELSA)^*^. *p < 0.05

Figure 2 shows the CLPMs for HL and depression in the eight Waves of the ELSA. The ICC was 0.51 showing a high degree of statistical dependence on the observations across the ELSA's repeated measures. The variance within people (across the repeated measures) was 0.12, representing small differences from Wave to Wave.

### Mediator

#### Quality of life

**Fig. 3:**
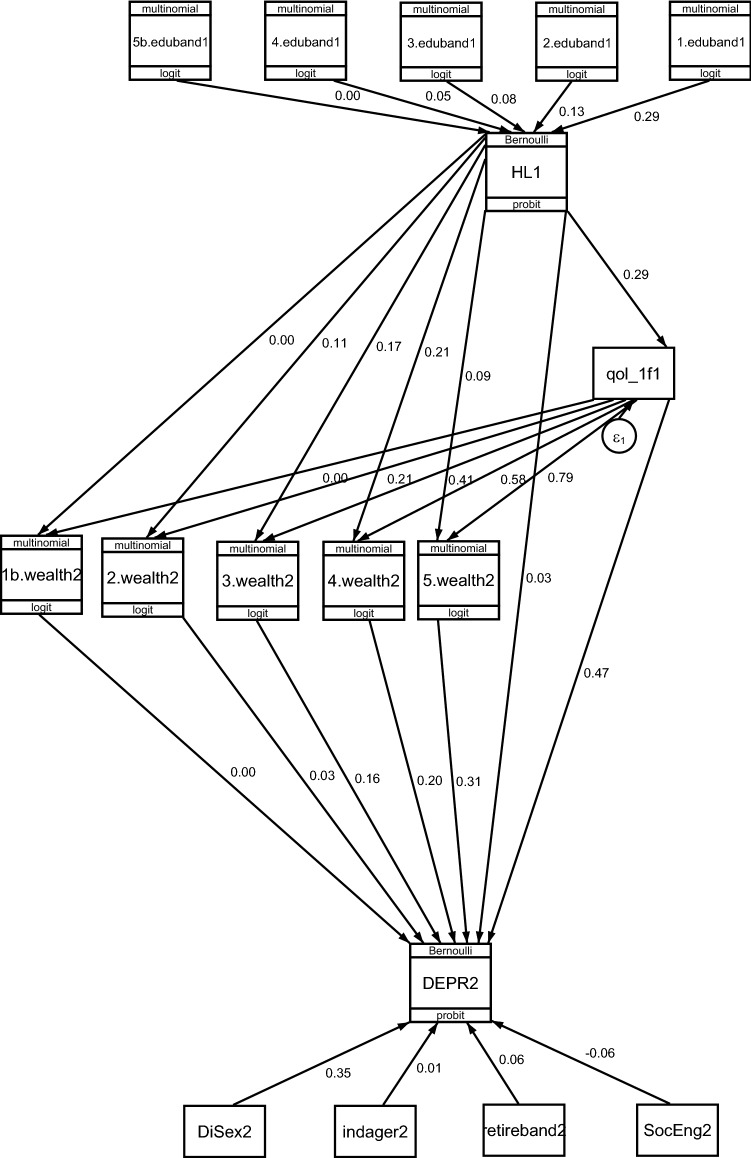
Standardised beta weights of the structural equation model representing the dynamic relationship between hearing loss in Wave 1, quality of life in Wave 1, socioeconomic position in Wave 2 and depression in Wave 2 of the English Longitudinal Study of Ageing (ELSA)*. *Exogenous variable eduband: representing the educational level in five categories (5: degree/higher education; 4: A level; 3: O levels CSE; 2: foreign/other; 1: no qualifications). HL: self-reported hearing loss in Wave 1; examined as an exogenous predictor that has a uni-directional effect on wealth. qol_1f1: CASP-19 confirmatory factor analyses factor score in Wave 1; functioned as an endogenous mediator variable that intervenes between HL and wealth, explaining the relation between HL and SEP. wealth2: socioeconomic position according to wealth in Wave 2; functioned both as an endogenous outcome variable and a moderating/intermediate dependent variable of depression (1 represents highest quintile; 5 represents lowest quintile). DEPR2: exogenous outcome variable; represents participants with CES-D Score ⩾4 in Wave 2. Control factors: DiSex: sex of study participants, indager2: age of the participants in Wave 2, retireband2: retirement status of the participants in Wave 2, SocEng2: social engagement in Wave 2

Results for principal component factor analysis (PCFA) and the factor scoring coefficients of the latent variables of quality of life for each Wave are included as Table 2 and Figs 1, 2, 3, 4, 5, 6, 7 8 of the supplementary material, respectively. The alpha reliability estimates for the CFA models ranged from 0.83 to 0.86, which is over the 0.70 minimum standard value [37], showing excellent reliability of the scales for the actual score of quality of life. Figure 3 depicts the dynamic CLPM model we constructed, and the standardised path coefficients of the effects of quality of life and wealth in the association between HL (Wave 1) with depression (Wave 2). The direct effect of HL on depression was weak. However, the relationship between HL and depression was temporally explained by the quality of life as a mediator. HL affected the different wealth groups disproportionally, mediated by the quality of life; Sobel's tests in all CLPMs indicated that there was variation in the role of quality of life across different wealth groups, for those with HL. In all Waves, those in the lowest wealth quintiles experienced over double the effect of quality of life compared to those in the highest wealth quintiles.

### Moderators

#### Socioeconomic position

Wealth moderated the effect of HL on depression. The effect of HL was stronger for those in the lowest wealth group (total effect: 0.72, interpreted as a correlation coefficient) compared to those in the highest, who were experiencing a more modest association between HL in Wave 1 and depression in Wave 2 (total effect: 0.48). Wealth moderated the association between HL and depression. In all Waves, those in the lowest wealth quintiles were experiencing strong/very strong association between HL and elevated depressive symptoms two years later (e.g. standardised β coefficient 0.72 to 0.98). In contrast, the association for those in the highest wealth quintiles was low to moderate (e.g. standardised β coefficient 0.48 to 0.58). The standardised mediation effects for each endogenous effect in the CLPMs and all CLPMs are presented as Table 3 and Figs. 9, 10, 11, 12, 13, 14, 15 of the supplementary material, respectively. In addition, the results of the subgroup analyses of the CLPMs are shown in Table 4 of the supplementary material. The self-reported measure of hearing in the ELSA underestimated the effect that HL has on SEP, showing a weak coefficient for all wealth groups. The improved self-reported definition of hearing data in Wave 7 [5] revealed a stronger association between HL and wealth, of similar strength to the association observed when using the objective measures of hearing in the ELSA Wave 7.

Regarding the magnitude of strength of the association of HL with depression, all measures showed a graded relationship; those in the lowest wealth quintile were experiencing a strong/very strong association between HL and elevated depressive symptoms, but the association in those in the highest wealth quintiles was moderate: (standardised β coefficient 0.89 versus 0.52 for self-reported, 0.98 versus 0.51 for improved self-reported and 0.86 versus 0.51 for the objective measure of HL, respectively).

#### Hearing aids use

The use of hearing aids moderated the effect of HL on depressive symptoms disproportionally, according to wealth; the moderation effect of hearing aids on depressive symptoms was higher for those in the lowest versus those in the highest wealth quintiles. The standardised β coefficient in those with self-reported HL in the ELSA Wave 7 became 0.56 from 0.89 for the lowest wealth quintile (improvement of effect: b = 0.33) while it remained stable in the highest wealth quintile. The improved self-reported measures of those with HL showed a slightly higher beneficial effect of hearing aids in the lowest wealth groups (improvement of effect: b = 0.40).

The results of sensitivity analyses according to the different hearing measures and the compliance rate of hearing aids use (most of the time/ some of the time) are included as Fig. 16 and Figs. 17, 18, 19 of the supplementary material, respectively. Intervention with hearing aids had a more significant effect in those with HL belonging to the least wealthy group than those in the highest wealth quintile. For example, for those with self-reported HL using hearing aids some of the time (17.15%, *n* = 566), the percentage of the moderating effect that hearing aids accounted for in alleviating the consequences of HL on depressive symptoms was 5.56% for those in the wealthier group. In comparison, the percentage was 13.56% if they belonged to the least wealthy group.

## Discussion

This study examined the psychosocial mechanisms that help explain the prospective relationship between HL and depression in older adults, which was previously unknown. We found that HL affected the different wealth groups disproportionally, mediated by the quality of life of individuals. Wealth moderated HL's effect on depression, determining the magnitude of their association, which was higher in the lowest wealth quintiles. Therefore, we suggest a graded relationship between HL and depression according to SEP, with those in the lowest wealth quintile having a higher risk for depression compared to those in the highest wealth quintile.

We also found that hearing aids usage potentially alleviated the depressive symptoms associated with HL. Those in the lowest versus the highest wealth quintiles experienced considerably more improvement in their psychosocial wellbeing after the use of hearing aids compared to those in the highest wealth quintiles, and the improvement was slightly greater among those with the most frequent use of hearing aids. The graded benefit from hearing aids was shown irrespective of the HL measure (self-reported or objective) used.

An important strength of the paper was the use of dynamic GSEMs as opposed to static ‘baseline–predicts–outcome’ methodologies, which have limitations when investigating variables that change over time with increasing age, such as HL and depression in later life. GSEMs combine the power and flexibility of both SEMs and generalised linear models, offering the opportunity to evaluate causal relationships within a unified modelling framework and calculate both direct and indirect effects of multiple interacting factors simultaneously, reaching a high predictive ability of the effects [38, 39]. Also, the CFA that generated a latent variable for quality of life in each Wave led to a strong predictive power in the theoretically causal associations between variables.

The recent systematic review and meta-analysis by Lawrence et al. [9], revealed that the existing evidence regarding the association between HL and depression is conflicting; our study adds to this body of knowledge by identifying the unrecognised effect of SEP modification, which distorts the HL's effect on depression. Previous studies did not focus on the modifiable factors linked to socioeconomic inequalities in hearing health, and research on this topic is scarce [7]. Only 5 of the 24 cross-sectional and 7 of the 11 cohort studies in the meta-analysis by Lawrence et al. [9] adjusted for the confounding influence of SEP covariates, which resulted in significant variance in the crude association between HL and depression across studies. It is worth mentioning that only the two studies that adjusted for a variety of SEP measures in their analyses [40, 41] did not find an association of HL with depression in older adults. Thus, our study adds to the literature by focusing primarily on the role of SEP, which we suggest may satisfactorily explain the causal, temporal and graded relationship between HL and depression over time, which differs according to people’s rank in the social hierarchy [14]

Similarly, the socioeconomic pattern we first identify to the benefit from hearing aids on psychosocial wellbeing among those with HL may also explain why no effect of hearing aids use was found in the recent meta-analysis by Lawrence et al.: [9] no previous study examined the role of hearing aids under a socioeconomic perspective, so as to be able to identify their effect heterogeneity according to SEP and to firmly conclude, therefore, about whether the use of hearing aids has any effect in alleviating the depressive symptoms among those with HL, and whether hearing aids played a more significant role in alleviating the negative impact of HL among those in the lower social strata. One possible explanation for the above finding might be that hearing aids probably acted as a support measure and aided the most vulnerable people who already lacked life opportunities compared to the most affluent, so they were able to take more control back and keep participating in society. This finding complements the results obtained by the PCFA for the examined 19-items of CASP-19, which revealed that the ‘Control’ domain of the CASP-19 Scale was the most impacted domain for those having HL in all eight Waves of the ELSA (Factors 1–4 in Table 2 of supplementary material).

Our study also has novel clinical implications, as it adds to the understanding of the interrelationship between HL and depression [2]. The early detection of HL by primary care professionals in routine assessments could help prevent or delay the onset of depression, particularly in lower wealth groups. Untreated HL may not only have negative consequences for people’s functional ability and mental wellbeing, but also to society as a whole, as our paper showed that HL may widen the socioeconomic inequalities through its graded impact on people’s wealth [42]. Taking SEP into account is considered an essential element of depression prevention strategies in the general population [43]. Our findings confirm that SEP is equally important for preventing depression in older people with HL.

Future research should examine common underlying factors among participants with similar wealth, which could lead to preventive psychological interventions, along with online and web-based interventions [32] for older adults with comorbid HL and depression. Large-scale RCTs are needed to guide clinical practice and investigate whether HL treatment could be a monotherapy treatment for depression or could be used as augmentation with antidepressant medications [44].

## Limitations

However, the findings must be interpreted considering several limitations. Our analyses, were inevitably restricted to include only the available variables in the ELSA datasets. Therefore, omitted variable bias may occur, as we never know whether all relevant predictors have been included in our models, and one of those left out may determine the value of an endogenous variable [45].

Another limitation is that the ELSA study concentrates on individuals living in private households; thus, individuals living in institutions such as residential and nursing homes are not included in the samples [23].

Moreover, the CES–D scale does not measure the duration of symptoms; therefore, *DSM* criteria for major or minor depression cannot be applied to these data. In addition, predicting the presence of clinically elevated depressive symptoms over time, as we did in our study, refers less directly to symptom severity [32].

We were aware that the self-reported data in the ELSA underestimated objectively measured hearing problems [5]. The sensitivity analysis we run to investigate potential differences in estimates of depression was possible only for ELSA Wave 7; this is the only ELSA Wave that includes both self-reported and objective hearing measures for the same participants [24]. Therefore, the error introduced by the self-reported measures is likely to reduce external validity, as people with depression may differentially report information on hearing status, and also their responses may be biased by cultural or population characteristics.

Another limitation was that the ELSA questionnaire does not contain any information on dementia diagnosis at any ELSA Wave, which may impact on the examined associations. There are few variables that offer an indirect cognitive assessment, such as numerical ability, word-finding (verbal fluency) and prospective memory; however, these are not recorded in all Waves. Therefore, we decided to avoid the measurement bias from missing data, and we did not include any of these variables in the structural equation modelling. The recently developed harmonised cognitive assessment protocol, part of the Healthy Cognitive Aging Project (HCAP) [46] aims to fill the aforementioned gap in the ELSA dataset, and discriminate between normal cognitive performance, cognitive impairment, and dementia status of the participants. Information from the ELSA-HCAP study is available from the ELSA Wave 9 and onwards and should be used in future studies to quantify the consequences of severe cognitive impairment for mental well-being.

## Conclusion

HL may place older adults at risk of developing significant depressive symptoms, with the lowest wealth groups experiencing up to double the relative risk for depression. The use of hearing aids reduces the risk of depression, suggesting that interventions with hearing aids could alleviate HL's psychological burden. Increasing the HL treatment rate could be one effective strategy for risk reduction of depression, given the high prevalence of HL in older age, and its low treatment levels. Early intervention requires early identification of HL that could mitigate HL's negative impact on psychosocial wellbeing and quality of life. Future studies should investigate the potential effectiveness of the inclusion of screening for HL in the routine geriatric assessment guidelines in the enhancement of psychosocial health of older adults, particularly those in lower wealth groups.

## Supplementary Information

Below is the link to the electronic supplementary material.

Supplementary file1 (DOCX 3247 KB)

## Data Availability

The English Longitudinal Study of Ageing dataset is publicly available via the UK Data Service (http://www.ukdataservice.ac.uk).

## Declarations

### Code availability

Statistical code is available from the corresponding author upon reasonable request at dialechti.tsimpida@manchester.ac.uk.
