# Supplementary material for: The dynamic relationship between hearing loss, quality of life, socioeconomic position and depression and the impact of hearing aids: answers from the English Longitudinal Study of Ageing (ELSA)
Source: Soc Psychiatry Psychiatr Epidemiol. 2021 Aug 12;57(2):353–62. doi: 10.1007/s00127-021-02155-0 (PMC8784360; doi:10.1007/s00127-021-02155-0)
Supplement: Supplementary file 1 — Supplementary file1 (DOCX 3247 KB) [file 127_2021_2155_MOESM1_ESM.docx]

**Supplementary Material**

**1. Number of Interviews by Waves in the English Longitudinal Study of Ageing (ELSA)**

|  | **Fieldwork Period** | **Total archived interviews** | **Core Member interviews** | **Partner interviews** |
| --- | --- | --- | --- | --- |
| **Wave 1** | March 2002 – March 2003 | 12,099 | 11,391 | 708 |
| **Wave 2** | June 2004 – July 2005 | 9,432 | 8,780 | 652 |
| **Wave 3** | May 2006 – August 2007 | 9,771 | 8,810 | 961 |
| **Wave 4** | May 2008 – July 2009 | 11,050 | 9,886 | 1,164 |
| **Wave 5** | June 2010 – July 2011 | 10,274 | 9,090 | 1,184 |
| **Wave 6** | May 2012 – June 2013 | 10,601 | 9,169 | 1,432 |
| **Wave 7** | June 2014 – May 2015 | 9,666 | 8,249 | 1,417 |
| **Wave 8** | May 2016 – June 2017 | 8,445 | 7,223 | 1,222 |

**2. Questions of the 8-item short version of the Center for Epidemiologic Studies Depression (CES-D) Scale:**

a) “Did you feel depressed”?

b) “Did you feel everything you did was an effort”?

c) “Was your sleep restless”?

d) “Were you happy”?

e) “Did you feel lonely”?

f) “Did you enjoy life”?

g) “Did you feel sad”?

h) “Were you unable to get going”?

**3. Scoring criteria of the 8-item short version of the Center for Epidemiologic Studies Depression (CES-D) Scale**: The lowest possible score was 0 (no symptoms of depression), and the highest possible score was 8 (the highest number of symptoms of depression. We utilised the cut point of the eight-item dichotomous response scale (greater than or equal to four symptoms on the Scale) to denote elevated depressive symptoms. This level has shown good predictive accuracy to define clinically significant depressive symptoms that indicate risk for adverse outcomes.

**4. The CASP-19 Scale questions of all domains:**

Control:

1) How often feels age prevents them from doing things they like

2) How often feels what happens to them is out of their control

3) How often feels free to plan for the future

4) How often feels left out of things

Autonomy:

5) How often can do the things they want to do

6) How often family responsibilities prevent them from doing things

7) How often feels they can please themselves what they do

8) How often feels their health stops them doing what they want to do

9) How often shortage of money stops them doing things

Self-realisation:

10) How often feels full of energy these days

11) How often chooses to do things they have never have done before

12) How often feels satisfied with the way their life has turned out

13) How often feels that life is full of opportunities

14) How often feels the future looks good to them

Pleasure:

15) How often look forward to each day

16) How often feels that their life has meaning

17) How often enjoys the things they do

18) How often enjoys being in the company of others

19) How often looks back on their life with a sense of happiness

**5. Scoring criteria of the** **CASP-19 Scale**: The summation of 19 items (with response options within a 4-point Likert scale ranging from 0, “never” to 3, “often”) yields a range from 0 to 57 for the total score of quality of life for each respondent. Higher scores indicate higher total levels of satisfaction of quality of life and in each Scale of control, autonomy, self-realisation and pleasure, respectively.

**6. Definition of net total non-pension wealth**

The value of the primary house minus the outstanding primary house mortgage, the value of savings and shares minus depts, and the value of other properties and businesses, also known as *the sum of net financial, physical and housing wealth*.

**7. The cut-off points for the wealth group definition were:**

Wave 1 (2002-3): Lowest -less than £22k, 2nd-between £22k-£132k, 3rd-between £132k-£229k, 4th-between £229k-£403k, Highest –More than £403k,

Wave 4 (2008-09): Lowest -less than £60k, 2nd-between £60k-£201k, 3rd-between £201k-£303k, 4th-between £303k-£496k, Highest –More than £496k,

Wave 8 (2016-17): Lowest -less than £71k, 2nd-between £71k-£210k, 3rd-between £210k-£354k, 4th-between £354k-£575k, Highest –More than £575k.

**8. Definition of social engagement**

A continuous measure of social engagement included in the analysis, derived from a set of eight binary variables, asking whether the respondent is a member of various civic and social organisations, including a political party, neighbourhood watch group, church or religious group, charitable association, educational or evening class, social club, sports club or exercise class, or any other organisation. The summation of 8 items yields a range from 0 to 8 for the total score of social engagement.

| **Table 1** Participants’ non-modifiable demographic factors, and prevalence of hearing loss and elevated depressive symptoms in 8 Waves of English Longitudinal Study of Ageing (ELSA) | | | | | | | | | | | | | | | | |
| --- | --- | --- | --- | --- | --- | --- | --- | --- | --- | --- | --- | --- | --- | --- | --- | --- |
| **GOR^a^** | **Hearing loss^b^** | **Depression^c^** | **Men** | **Women** | **Age**  **50-64** | **Age**  **65-74** | **Age**  **75-89** | **Mean age (SD)^d^** | **Hearing loss^b^** | **Depression^c^** | **Men** | **Women** | **Age**  **50-64** | **Age**  **65-74** | **Age**  **75-89** | **Mean age (SD)** |
| **WAVE 1 (2002-2003)** | | | | | | | | | **WAVE 2 (2004-2005)** | | | | | | | |
| North East | 325 (42.54) | 151 (19.53) | 323  (41.79) | 450  (58.21) | 366  (49.80) | 245  (33.33) | 124  (16.87) | 64.33  (10.53) | 254  (43.34) | 98 (16.53) | 251  (42.33) | 342  (57.67) | 272  (47.06) | 189  (32.70) | 117  (20.24) | 65.79  (9.97) |
| North West | 603  (38.78) | 295  (18.71) | 692  (43.88) | 885  (56.12) | 799  (53.30) | 410  (27.35) | 290  (19.35) | 64.24  (10.95) | 432  (36.86) | 215 (18.02) | 517  (43.34) | 676  (56.66) | 551  (48.25) | 337  (29.51) | 254  (22.24) | 66.21  (10.91) |
| Yorkshire  and The Humber | 549  (43.06) | 245  (18.93) | 573  (44.28) | 721  (55.72) | 646  (52.73) | 351  (28.65) | 228  (18.61) | 63.84  (10.91) | 401  (40.46) | 148  (14.74) | 438  (43.63) | 566  (56.37) | 474  (48.77) | 294  (30.25) | 204  (20.99) | 65.57  (10.39) |
| East Midlands | 449  (39.18) | 173  (14.81) | 528  (45.21) | 640  (54.79) | 605  (55.50) | 297  (27.25) | 188  (17.25) | 63.30  (11.06) | 335  (35.52) | 146  (15.26) | 425  (44.41) | 532  (55.59) | 481  (52.17) | 247  (26.79) | 194  (21.04) | 64.99  (10.42) |
| West Midlands | 452  (36.78) | 210  (16.68) | 550  (43.69) | 709  (56.31) | 599  (49.92) | 358  (29.83) | 243  (20.25) | 64.77  (10.99) | 375  (38.82) | 146 (14.97) | 422  (43.28) | 553  (56.72) | 445  (47.34) | 290  (30.85) | 205  (21.81) | 66.06  (10.64) |
| East of England | 507  (37.50) | 169  (12.46) | 596  (43.95) | 760  (56.05) | 663  (51.88) | 368  (28.79) | 247  (19.33) | 64.19  (11.16) | 400  (37.00) | 131 (11.96) | 491  (44.84) | 604  (55.16) | 507  (48.33) | 317  (30.22) | 225  (21.45) | 65.81  (10.85) |
| London | 385  (34.16) | 203  (17.74) | 484  (42.31) | 660  (57.69) | 574  (53.75) | 268  (25.09) | 226  (21.16) | 64.46  (11.54) | 292  (35.39) | 159 (19.06) | 347  (41.61) | 487  (58.39) | 406  (50.94) | 222  (27.85) | 169  (21.20) | 66.05  (11.22) |
| South East | 681  (36.13) | 250  (13.08) | 823  (43.04) | 1089  (56.96) | 951  (52.57) | 484  (26.76) | 374  (20.67) | 64.27  (10.97) | 513  (35.19) | 201 (13.63) | 628  (42.58) | 847  (57.42) | 688  (48.38) | 391  (27.50) | 343  (24.12) | 66.14  (10.79) |
| South West | 510  (38.37) | 199  (14.82) | 602  (44.83) | 741  (55.17) | 605  (47.79) | 363  (28.67) | 298  (23.54) | 65.12  (11.38) | 420  (39.77) | 165 (15.48) | 468  (43.90) | 598  (56.10) | 462  (44.90) | 307  (29.83) | 260  (25.27) | 66.64  (10.61) |
| **WAVE 3 (2006-2007)** | | | | | | | | | **WAVE 4 (2008-2009)** | | | | | | | |
| North East | 284  (47.81) | 102 (16.86) | 259  (42.81) | 346  (57.19) | 289  (49.49) | 160  (27.40) | 135  (23.12) | 65.51  (10.67) | 284  (47.81) | 97 (14.85) | 274  (41.96) | 379  (58.04) | 306  (48.19) | 185  (29.13) | 144  (22.68) | 66.53  (10.50) |
| North West | 458  (40.39) | 177 (15.25) | 508  (43.76) | 653  (56.24) | 593  (54.45) | 276  (25.34) | 220  (20.20) | 64.53  (11.41) | 458  (40.39) | 197 (14.95) | 586  (44.46) | 732  (55.54) | 682  (53.41) | 352  (27.56) | 243  (19.03) | 65.15  (10.57) |
| Yorkshire  and The Humber | 452  (42.68) | 188 (17.30) | 475  (43.70) | 612  (56.30) | 546  (53.37) | 275  (26.88) | 202  (19.75) | 64.16  (11.16) | 452  (42.68) | 168 (14.65) | 492  (42.89) | 655  (57.11) | 573  (52.04) | 322  (29.25) | 206  (18.71) | 64.95  (10.41) |
| East Midlands | 368  (37.98) | 167 (16.87) | 440  (44.44) | 550  (55.56) | 537  (57.19) | 227  (24.17) | 175  (18.64) | 63.43  (10.95) | 368  (37.98) | 182 (16.26) | 496  (44.36) | 622  (55.64) | 597  (55.28) | 294  (27.22) | 189  (17.50) | 64.88  (10.34) |
| West Midlands | 392  (39.68) | 155  (15.42) | 435  (43.28) | 570  (56.72) | 503  (52.89) | 243  (25.55) | 205  (21.56) | 65.13  (11.64) | 392  (39.68) | 179 (15.55) | 511  (44.40) | 640  (55.60) | 565  (50.81) | 314  (28.24) | 233  (20.95) | 66.00  (10.71) |
| East of England | 461  (40.33) | 141 (12.07) | 524  (44.86) | 644  (55.14) | 585  (52.89) | 297  (26.85) | 224  (20.25) | 64.42  (11.48) | 461  (40.33) | 168 (12.70) | 596  (45.05) | 727  (54.95) | 667  (52.73) | 391  (30.91) | 207  (16.36) | 64.96  (10.44) |
| London | 348  (40.65) | 158 (18.10) | 356  (40.78) | 517  (59.22) | 451  (55.41) | 199  (24.45) | 164  (20.15) | 64.77  (12.06) | 348  (40.65) | 164 (17.63) | 404  (43.44) | 526  (56.56) | 487  (54.90) | 242  (27.28) | 158  (17.81) | 65.44  (11.09) |
| South East | 534  (35.77) | 172 (11.21) | 654  (42.63) | 880  (57.37) | 767  (52.86) | 371  (25.57) | 313  (21.57) | 64.84  (11.42) | 534  (35.77) | 218 (12.14) | 781  (43.51) | 1014  (56.49) | 902  (52.17) | 496  (28.69) | 331  (19.14) | 65.28  (10.35) |
| South West | 453  (43.06) | 142 (13.31) | 476  (44.61) | 591  (55.39) | 495  (48.82) | 266  (26.23) | 253  (24.95) | 65.81  (11.51) | 453  (43.06) | 156 (12.97) | 530  (44.06) | 673  (55.94) | 561  (48.32) | 351  (30.23) | 249  (21.45) | 66.14  (10.54) |
| **WAVE 5 (2010-2011)** | | | | | | | | | **WAVE 6 (2012-2013)** | | | | | | | |
| North East | 295  (51.85) | 87 (14.75) | 255  (43.29) | 334  (56.71) | 241  (41.70) | 177  (30.62) | 160  (27.68) | 66.78  (12.69) | 249  (50.00) | 65 (12.48) | 226  (43.38) | 295  (56.62) | 185  (36.42) | 167  (32.87) | 156  (30.71) | 66.97  (14.74) |
| North West | 481  (42.68) | 199 (16.79) | 531  (44.81) | 654  (55.19) | 575  (49.74) | 338  (29.24) | 243  (21.02) | 65.03  (12.99) | 471  (46.96) | 160 (14.97) | 481  (45.00) | 588  (55.00) | 442  (42.14) | 359  (34.22) | 248  (23.64) | 66.61  (12.61) |
| Yorkshire  and The Humber | 457  (45.11) | 146 (13.98) | 442  (42.34) | 602  (57.66) | 484  (47.78) | 315  (31.10) | 214  (21.13) | 65.29  (12.26) | 453  (48.81) | 131 (13.53) | 407  (42.05) | 561  (57.95) | 401  (42.57) | 305  (32.38) | 236  (25.05) | 66.58  (12.54) |
| East Midlands | 399  (39.66) | 172 (16.27) | 467  (44.18) | 590  (55.82) | 522  (50.68) | 297  (28.83) | 211  (20.49) | 64.85  (12.34) | 391  (41.68) | 147 (14.91) | 430  (43.61) | 556  (56.39) | 406  (42.34) | 333  (34.72) | 220  (22.94) | 65.86  (13.36) |
| West Midlands | 413  (40.73) | 179 (16.73) | 484  (45.23) | 586  (54.77) | 461  (44.93) | 333  (32.46) | 232  (22.61) | 64.45  (15.38) | 399  (42.95) | 152 (15.54) | 436  (44.58) | 542  (55.42) | 354  (37.78) | 323  (34.47) | 260  (27.75) | 65.58  (16.05) |
| East of England | 503  (42.20) | 180 (14.50) | 556  (44.80) | 685  (55.20) | 556  (46.37) | 384  (32.03) | 259  (21.60) | 65.28  (13.34) | 514  (46.77) | 109 (9.52) | 504  (44.02) | 641  (55.98) | 438  (39.75) | 393  (35.66) | 271  (24.59) | 65.74  (14.78) |
| London | 333  (40.71) | 142 (16.59) | 349  (40.77) | 507  (59.23) | 404  (49.75) | 238  (29.31) | 170  (20.94) | 63.54  (15.57) | 302  (41.26) | 105 (13.64) | 322  (41.82) | 448  (58.18) | 314  (42.43) | 255  (34.46) | 171  (23.11) | 65.08  (15.46) |
| South East | 615  (39.55) | 184 (11.34) | 697  (42.97) | 925  (57.03) | 739  (46.89) | 490  (31.09) | 347  (22.02) | 65.46  (13.06) | 639  (44.53) | 151 (10.07) | 648  (43.23) | 851  (56.77) | 569  (38.89) | 523  (35.75) | 371  (25.36) | 66.45  (13.40) |
| South West | 469  (42.95) | 139 (12.14) | 504  (44.02) | 641  (55.98) | 489  (44.01) | 354  (31.86) | 268  (24.12) | 66.14  (13.12) | 489  (48.71) | 98  (9.26) | 476  (44.99) | 582  (55.01) | 378  (37.06) | 361  (35.39) | 281  (27.55) | 66.28  (15.16) |
| **WAVE 7 (2014-2015)** | | | | | | | | | **WAVE 8 (2016-2017)** | | | | | | | |
| North East | 212  (50.12) | 48 (11.01) | 195  (43.43) | 254  (56.57) | 132  (30.28) | 158  (51.92) | 146  (33.49) | 68.10  (15.45) | 202  (53.72) | 51 (12.81) | 172  (43.22) | 226  (56.78) | 82  (20.97) | 161  (41.18) | 148  (37.85) | 70.39  (13.04) |
| North West | 378  (43.15) | 127 (14.05) | 402  (43.41) | 524  (56.59) | 308  (34.07) | 365  (61.21) | 231  (25.55) | 67.15  (13.72) | 370  (48.49) | 95 (11.89) | 349  (43.68) | 450  (56.32) | 205  (26.42) | 357  (46.01) | 214  (27.58) | 68.02  (14.45) |
| Yorkshire  and The Humber | 360  (46.51) | 100 (12.61) | 339  (42.06) | 467  (57.94) | 262  (33.04) | 318  (59.85) | 213  (26.86) | 68.19  (11.71) | 339  (48.36) | 111 (15.14) | 302  (41.20) | 431  (58.80) | 188  (26.18) | 310  (43.18) | 220  (30.64) | 69.04  (12.69) |
| East Midlands | 351  (42.39) | 115 (13.61) | 374  (43.19) | 492  (56.81) | 296  (35.03) | 327  (59.52) | 222  (26.27) | 67.51  (13.18) | 351  (47.56) | 99 (12.87) | 331  (43.04) | 438  (56.96) | 204  (27.20) | 330  (44.00) | 216  (28.80) | 68.64  (13.53) |
| West Midlands | 359  (44.65) | 117 (14.18) | 390  (45.56) | 466  (54.44) | 274  (33.21) | 299  (54.23) | 252  (30.55) | 66.78  (16.46) | 340  (48.02) | 108 (14.32) | 346  (45.89) | 408  (54.11) | 203  (28.04) | 294  (40.61) | 227  (31.35) | 67.52  (16.94) |
| East of England | 417  (42.55) | 100 (10.09) | 448  (43.62) | 579  (56.38) | 324  (32.69) | 383  (57.39) | 284  (28.66) | 67.24  (14.55) | 423  (47.37) | 78  (8.41) | 402  (43.37) | 525  (56.63) | 218  (24.17) | 385  (42.68) | 299  (33.15) | 68.81  (14.20) |
| London | 258  (40.12) | 94 (14.42) | 284  (42.07) | 391  (57.93) | 239  (36.66) | 241  (58.30) | 172  (26.38) | 66.50  (15.04) | 249  (46.03) | 75 (13.18) | 239  (42.00) | 330  (58.00) | 158  (28.42) | 223  (40.11) | 175  (31.47) | 68.83  (13.78) |
| South East | 537  (42.96) | 131 (10.23) | 551  (41.90) | 764  (58.10) | 388  (30.29) | 512  (57.32) | 381  (29.74) | 67.85  (13.94) | 508  (46.35) | 121 (10.58) | 479  (41.87) | 665  (58.13) | 253  (22.71) | 499  (44.79) | 362  (32.50) | 69.17  (14.36) |
| South West | 413  (46.67) | 88  (9.71) | 411  (43.82) | 527  (56.18) | 279  (30.79) | 348  (55.48) | 279  (30.79) | 67.59  (15.39) | 399  (52.02) | 85 (10.64) | 344  (43.05) | 455  (56.95) | 175  (22.58) | 349  (45.03) | 251  (32.39) | 68.79  (15.24) |

Values are expressed as column N (%) unless otherwise is indicated.

^a^ GOR: Government Office Regions

^b^ Self-reported hearing loss: the sum of those that rated their hearing as fair or poor on a five-point Likert scale (excellent, very good, good, fair or poor), or responded positively in the question whether they find it difficult to follow a conversation if there is background noise (such as TV, radio or children playing).

^c^ Elevated depressive symptoms: the cut point of the eight-item dichotomous response scale (greater than or equal to four symptoms on the Scale) (8CES-D⩾4).

^d^ Mean (SD): mean age in years (Standard deviation)

|  | **Eigenvalue for each factor and Cumulative proportion of variance** | | | | | | | | | | | | | | | |
| --- | --- | --- | --- | --- | --- | --- | --- | --- | --- | --- | --- | --- | --- | --- | --- | --- |
| **Factor** | **Wave 1** | **Cum.*** | **Wave 2** | **Cum.*** | **Wave 3** | **Cum.*** | **Wave 4** | **Cum.*** | **Wave 5** | **Cum.*** | **Wave 6** | **Cum.*** | **Wave 7** | **Cum.*** | **Wave 8** | **Cum.*** |
| Factor 1 | 5.55 | 0.29 | 5.77 | 0.30 | 5.79 | 0.30 | 5.92 | 0.31 | 6.01 | 0.32 | 6.05 | 0.32 | 6.08 | 0.32 | 6.06 | 0.31 |
| Factor 2 | 1.95 | 0.39 | 2.01 | 0.40 | 1.98 | 0.40 | 1.97 | 0.41 | 2.00 | 0.43 | 2.03 | 0.43 | 2.06 | 0.43 | 2.05 | 0.43 |
| Factor 3 | 1.25 | 0.46 | 1.26 | 0.48 | 1.18 | 0.47 | 1.15 | 0.48 | 1.16 | 0.49 | 1.14 | 0.49 | 1.20 | 0.49 | 1.16 | 0.49 |
| Factor 4 | 1.15 | 0.52 | 1.18 | 0.54 | 1.10 | 0.53 | 1.11 | 0.53 | 1.07 | 0.54 | 1.09 | 0.54 | 1.09 | 0.55 | 1.09 | 0.55 |
| Factor 5 | 0.96 | 0.57 | 0.95 | 0.59 | 0.89 | 0.58 | 0.91 | 0.58 | 0.92 | 0.59 | 0.93 | 0.59 | 0.93 | 0.60 | 0.95 | 0.60 |
| Factor 6 | 0.89 | 0.62 | 0.89 | 0.64 | 0.97 | 0.62 | 0.88 | 0.63 | 0.89 | 0.64 | 0.89 | 0.64 | 0.85 | 0.64 | 0.87 | 0.64 |
| Factor 7 | 0.80 | 0.66 | 0.77 | 0.68 | 0.83 | 0.67 | 0.80 | 0.67 | 0.79 | 0.68 | 0.80 | 0.68 | 0.80 | 0.69 | 0.78 | 0.68 |
| Factor 8 | 0.75 | 0.70 | 0.74 | 0.71 | 0.76 | 0.71 | 0.75 | 0.71 | 0.74 | 0.72 | 0.72 | 0.72 | 0.74 | 0.72 | 0.75 | 0.72 |
| Factor 9 | 0.70 | 0.74 | 0.67 | 0.75 | 0.70 | 0.74 | 0.69 | 0.74 | 0.69 | 0.76 | 0.66 | 0.75 | 0.66 | 0.76 | 0.74 | 0.76 |
| Factor 10 | 0.67 | 0.77 | 0.65 | 0.78 | 0.68 | 0.78 | 0.65 | 0.78 | 0.66 | 0.79 | 0.63 | 0.79 | 0.64 | 0.79 | 0.62 | 0.79 |
| Factor 11 | 0.62 | 0.81 | 0.61 | 0.81 | 0.63 | 0.81 | 0.63 | 0.81 | 0.61 | 0.82 | 0.59 | 0.82 | 0.60 | 0.82 | 0.59 | 0.82 |
| Factor 12 | 0.59 | 0.84 | 0.58 | 0.85 | 0.58 | 0.84 | 0.59 | 0.84 | 0.56 | 0.85 | 0.56 | 0.85 | 0.54 | 0.85 | 0.55 | 0.85 |
| Factor 13 | 0.55 | 0.87 | 0.52 | 0.87 | 0.54 | 0.87 | 0.52 | 0.87 | 0.50 | 0.88 | 0.51 | 0.88 | 0.52 | 0.88 | 0.50 | 0.88 |
| Factor 14 | 0.51 | 0.89 | 0.50 | 0.90 | 0.50 | 0.89 | 0.49 | 0.90 | 0.47 | 0.90 | 0.49 | 0.90 | 0.47 | 0.90 | 0.48 | 0.90 |
| Factor 15 | 0.47 | 0.91 | 0.44 | 0.92 | 0.46 | 0.92 | 0.45 | 0.92 | 0.43 | 0.93 | 0.44 | 0.93 | 0.42 | 0.93 | 0.44 | 0.92 |
| Factor 16 | 0.44 | 0.94 | 0.43 | 0.95 | 0.42 | 0.94 | 0.43 | 0.95 | 0.40 | 0.95 | 0.42 | 0.95 | 0.40 | 0.95 | 0.42 | 0.95 |
| Factor 17 | 0.41 | 0.96 | 0.36 | 0.97 | 0.38 | 0.96 | 0.37 | 0.97 | 0.36 | 0.97 | 0.36 | 0.97 | 0.34 | 0.97 | 0.36 | 0.97 |
| Factor 18 | 0.40 | 0.98 | 0.35 | 0.98 | 0.37 | 0.98 | 0.34 | 0.98 | 0.34 | 0.98 | 0.34 | 0.98 | 0.33 | 0.98 | 0.35 | 0.98 |
| Factor 19 | 0.31 | 1.00 | 0.30 | 1.00 | 0.30 | 1.00 | 0.29 | 1.00 | 0.29 | 1.00 | 0.29 | 1.00 | 0.30 | 1.00 | 0.30 | 1.00 |
| Number of obs. | 9,723 |  | 7,193 |  | 7,561 |  | 8,595 |  | 8,299 |  | 7,237 |  | 6,263 |  | 5,580 |  |
| Retained factors | 4 |  | 4 |  | 4 |  | 4 |  | 4 |  | 4 |  | 4 |  | 4 |  |
| Alpha reliability | 0.83 |  | 0.85 |  | 0.85 |  | 0.85 |  | 0.86 |  | 0.85 |  | 0.86 |  | 0.85 |  |

**Table 2. Results of the principal component factor analysis (PCFA) for the 19-items of CASP-19 in 8 Waves of ELSA**

*Cumulative proportion of variance of the 19-items in each Wave

| **Table 3**. Standardised effects of the generalised structural equation modelling (GSEM) mediation analyses of 74,908 person-years in the 8 Waves of English Longitudinal Study of Ageing (ELSA) (full details in the footnote) | | | | | |
| --- | --- | --- | --- | --- | --- |
| **Outcome** | **Direct effect^^[[1]](#endnote-1)^^** | **Indirect effect** | **Total effect** | **Sobel Test^^[[2]](#endnote-2)^^** | **% of effect mediated ^^[[3]](#endnote-3)^^** |
| **Cross-lagged model^^[[4]](#endnote-4)^^ path No1** | | | | | |
| **QoL^^[[5]](#endnote-5)^^ at Wave 1** | | | | | |
| HL^^[[6]](#endnote-6)^^1->QoL1^^[[7]](#endnote-7)^^ | 0.29 | | | | |
| **SEP ^^[[8]](#endnote-8)^^ at Wave 2 as mediated by the quality of life** | | | | | |
| HL1->1wealth2^^[[9]](#endnote-9)^^ | (Reference) | | | | |
| HL1->2wealth2 | 0.11 | 0.06 | 0.17 | 4.94 | 35.29 |
| HL1->3wealth2 | 0.17 | 0.12 | 0.29 | 8.40 | 41.38 |
| HL1->4wealth2 | 0.21 | 0.17 | 0.38 | 10.25 | 44.74 |
| HL1->5wealth2 | 0.09 | 0.23 | 0.32 | 11.69 | 71.88 |
| **DEPR^^[[10]](#endnote-10)^^ at Wave 2 due to HL, according to wealth (quintiles)** | | | | | |
| HL1->DEPR2 | 0.03 | | | | |
| QoL1(HL1)->DEPR2 for 1wealth2 |  | (Reference) | | | |
| QoL1(HL1)->DEPR2 for 2wealth2 | 0.47  (direct effect) | 0.01 | 0.48 | 0.42 | 2.08 |
| QoL1(HL1)->DEPR2 for 3wealth2 |  | 0.07 | 0.54 | 2.58 | 12.96 |
| QoL1(HL1)->DEPR2 for 4wealth2 |  | 0.12 | 0.59 | 2.80 | 20.34 |
| QoL1(HL1)->DEPR2 for 5wealth2 |  | 0.25 | 0.72 | 4.32 | 34.72 |
| **Cross-lagged model path No2** | | | | | |
| **QoL at Wave 3** | | | | | |
| HL3->QoL3 | 0.29 | | | | |
| **SEP at Wave 4 as mediated by the quality of life** | | | | | |
| HL3->1wealth4 | (Reference) | | | | |
| HL3->2wealth4 | -0.07 | 0.05 | -0.02 | 4.08 | -250.00 |
| HL3->3wealth4 | 0.12 | 0.10 | 0.22 | 7.49 | 45.45 |
| HL3->4wealth4 | 0.07 | 0.14 | 0.21 | 8.12 | 66.67 |
| HL3->5wealth4 | -0.09 | 0.24 | 0.15 | 10.86 | 160.00 |
| **DEPR at Wave 4 due to HL, according to wealth (quintiles)** | | | | | |
| HL3->DEPR4 | 0.13 | | | | |
| QoL3(HL3)->DEPR4 for 1wealth4 |  | (Reference) | | | |
| QoL3(HL3)->DEPR4 for 2wealth4 | 0.54  (direct effect) | 0.03 | 0.57 | 1.72 | 5.26 |
| QoL3(HL3)->DEPR4 for 3wealth4 |  | 0.05 | 0.59 | 1.58 | 8.47 |
| QoL3(HL3)->DEPR4 for 4wealth4 |  | 0.14 | 0.68 | 3.40 | 20.59 |
| QoL3(HL3)->DEPR4 for 5wealth4 |  | 0.28 | 0.82 | 4.11 | 34.15 |
| **Cross-lagged model path No3** | | | | | |
| **QoL at Wave 5** | | | | | |
| HL5->QoL5 | 0.29 | | | | |
| **SEP at Wave 6 as mediated by the quality of life** | | | | | |
| HL5->1wealth6 | (Reference) | | | | |
| HL5->2wealth6 | -0.06 | 0.07 | 0.01 | 4.73 | 700.00 |
| HL5->3wealth6 | -0.02 | 0.14 | 0.12 | 9.36 | 116.67 |
| HL5->4wealth6 | 0.11 | 0.20 | 0.31 | 11.10 | 64.52 |
| HL5->5wealth6 | -0.10 | 0.26 | 0.25 | 11.34 | 104.00 |
| **DEPR at Wave 6 due to HL, according to wealth (quintiles)** | | | | | |
| HL5->DEPR6 | 0.12 | | | | |
| QoL5(HL5)->DEPR6 for 1wealth6 |  | (Reference) |  |  |  |
| QoL5(HL5)->DEPR6 for 2wealth6 | 0.53  (direct effect) | 0.01 | 0.54 | -0.62 | 1.85 |
| QoL5(HL5)->DEPR6 for 3wealth6 |  | 0.07 | 0.60 | 1.85 | 11.67 |
| QoL5(HL5)->DEPR6 for 4wealth6 |  | 0.21 | 0.74 | 3.66 | 28.38 |
| QoL5(HL5)->DEPR6 for 5wealth6 |  | 0.35 | 0.88 | 4.70 | 39.77 |
| (Continued) | | | | | |
|  | | | | | |
|  | | | | | |
|  | | | | | |
| **Cross-lagged model path No4** | | | | | |
| **QoL at Wave 7** | | | | | |
| HL7->QoL7 | 0.26 | | | | |
| **SEP at Wave 8 as mediated by the quality of life** | | | | | |
| HL7->1wealth8 | (Reference) | | | | |
| HL7->2wealth8 | 0.00 | 0.05 | 0.05 | 3.63 | 100.00 |
| HL7->3wealth8 | -0.09 | 0.12 | 0.03 | 6.31 | 400.00 |
| HL7->4wealth8 | 0.07 | 0.16 | 0.23 | 7.14 | 69.57 |
| HL7->5wealth8 | -0.12 | 0.22 | 0.10 | 7.70 | 220.00 |
| **DEPR at Wave 8 due to HL, according to wealth (quintiles)** | | | | | |
| HL7->DEPR8 | 0.15 | | | | |
| QoL7(HL8)->DEPR8 for 1wealth8 |  | (Reference) |  |  |  |
| QoL7(HL8)->DEPR8 for 2wealth8 | 0.51  (direct effect) | 0.01 | 0.52 | 0.65 | 1.92 |
| QoL7(HL8)->DEPR8 for 3wealth8 |  | 0.14 | 0.65 | 3.23 | 21.54 |
| QoL7(HL8)->DEPR8 for 4wealth8 |  | 0.18 | 0.69 | 3.02 | 26.09 |
| QoL7(HL8)->DEPR8 for 5wealth8 |  | 0.38 | 0.89 | 4.24 | 42.70 |
| **Cross-lagged model path No5** | | | | | |
| **QoL at Wave 2** | | | | | |
| HL2->QoL2 | 0.26 | | | | |
| **SEP at Wave 3 as mediated by the quality of life** | | | | | |
| HL2->1wealth3 | (Reference) | | | | |
| HL2->2wealth3 | 0.02 | 0.08 | 0.10 | 5.36 | 80.00 |
| HL2->3wealth3 | 0.12 | 0.13 | 0.25 | 7.69 | 52.00 |
| HL2->4wealth3 | 0.17 | 0.18 | 0.35 | 9.45 | 51.43 |
| HL2->5wealth3 | 0.01 | 0.24 | 0.25 | 10.81 | 96.00 |
| **DEPR at Wave 3 due to HL, according to wealth (quintiles)** | | | | | |
| HL2->DEPR3 | 0.17 | | | | |
| QoL2(HL2)->DEPR3 for 1wealth3 |  | (Reference) |  |  |  |
| QoL2(HL2)->DEPR3 for 2wealth3 | 0.50  (direct effect) | 0.07 | 0.57 | 2.77 | 12.28 |
| QoL2(HL2)->DEPR3 for 3wealth3 |  | 0.16 | 0.66 | 4.09 | 24.24 |
| QoL2(HL2)->DEPR3 for 4wealth3 |  | 0.29 | 0.79 | 5.31 | 36.71 |
| QoL2(HL2)->DEPR3 for 5wealth3 |  | 0.48 | 0.98 | 5.84 | 48.98 |
| **Cross-lagged model path No6** | | | | | |
| **QoL at Wave 4** | | | | | |
| HL4->QoL4 | 0.25 | | | | |
| **SEP at Wave 5 as mediated by the quality of life** | | | | | |
| HL4->1wealth5 | (Reference) | | | | |
| HL4->2wealth5 | -0.03 | 0.07 | 0.04 | 5.77 | 175.00 |
| HL4->3wealth5 | 0.06 | 0.09 | 0.15 | 7.56 | 60.00 |
| HL4->4wealth5 | 0.04 | 0.16 | 0.20 | 9.85 | 80.00 |
| HL4->5wealth5 | -0.03 | 0.20 | 0.17 | 10.64 | 117.65 |
| **DEPR at Wave 5 due to HL, according to wealth (quintiles)** | | | | | |
| HL4->DEPR5 | 0.19 | | | | |
| QoL4(HL4)->DEPR5 for 1wealth5 |  | (Reference) |  |  |  |
| QoL4(HL4)->DEPR5 for 2wealth5 | 0.50  (direct effect) | 0.00 | 0.50 | 0 | 0 |
| QoL4(HL4)->DEPR5 for 3wealth5 |  | 0.02 | 0.52 | 0.85 | 3.85 |
| QoL4(HL4)->DEPR5 for 4wealth5 |  | 0.17 | 0.67 | 3.62 | 25.37 |
| QoL4(HL4)->DEPR5 for 5wealth5 |  | 0.32 | 0.82 | 5.50 | 39.02 |
| (Continued) | | | | | |
|  | | | | | |
|  | | | | | |
|  | | | | | |
| **Cross-lagged model path No7** | | | | |  |
| **QoL at Wave 6** | | | | | |
| HL6->QoL6 | 0.22 | | | | |
| **SEP at Wave 7 as mediated by the quality of life** | | | | | |
| HL6->1wealth7 | (Reference) | | | | |
| HL6->2wealth7 | -0.04 | 0.04 | 0 | 3.76 | 0 |
| HL6->3wealth7 | -0.07 | 0.09 | -0.02 | 6.68 | -450.00 |
| HL6->4wealth7 | -0.06 | 0.14 | 0.08 | 8.23 | 175.00 |
| HL6->5wealth7 | -0.23 | 0.18 | -0.05 | 9.20 | -360.00 |
| **DEPR at Wave 7 due to HL, according to wealth (quintiles)** | | | | | |
| HL6->DEPR7 | 0.20 | | | | |
| QoL6(HL6)->DEPR3 for 1wealth7 |  | (Reference) |  |  |  |
| QoL6(HL6)->DEPR3 for 2wealth7 | 0.56  (direct effect) | 0.02 | 0.58 | 0.97 | 3.45 |
| QoL6(HL6)->DEPR3 for 3wealth7 |  | 0.05 | 0.61 | 1.32 | 8.20 |
| QoL6(HL6)->DEPR3 for 4wealth7 |  | 0.23 | 0.79 | 3.90 | 29.11 |
| QoL6(HL6)->DEPR3 for 5wealth7 |  | 0.36 | 0.92 | 4.60 | 39.13 |

| **Table 4**. Standardised effects of the generalised structural equation modelling (GSEM) mediation analyses according to different hearing loss measures in Waves 7 and 8 of the English Longitudinal Study of Ageing (ELSA) and the effect of intervention with hearing aids (full details in the footnote) | | | | | | | | | | | | | | | |
| --- | --- | --- | --- | --- | --- | --- | --- | --- | --- | --- | --- | --- | --- | --- | --- |
| **Outcome** | **Direct effect^^[[11]](#endnote-11)^^** | **Indirect effect** | **Total effect** | **Sobel Test^^[[12]](#endnote-12)^^** | **% of**  **effect mediated ^^[[13]](#endnote-13)^^** | **Direct effect** | **Indirect effect** | **Total effect** | **Sobel Test** | **% of effect mediated** | **Direct effect** | **Indirect effect** | **Total effect** | **Sobel Test** | **% of effect mediated** |
| **Self-reported HL*** | | | | | | **Self-reported HL (improved measures**)** | | | | | **Objective HL (via HearCheck Screener)***** | | | | |
| **QoL at Wave 7** | | | | | |  | | | | |  | | | | |
| HL7->QoL7 | 0.26 | | | | | 0.25 | | | | | 0.20 | | | | |
| **SEP at Wave 8 as mediated by the quality of life** | | | | | |  | | | | |  | | | | |
| HL7->1wealth8 | (Reference) | | | | | (Reference) | | | | | (Reference) | | | | |
| HL7->2wealth8 | 0.00 | 0.05 | 0.05 | 3.63 | 100.00 | 0.19 | 0.01 | 0.20 | 0.50 | 5.00 | 0.35 | 0.04 | 0.39 | 3.30 | 10.26 |
| HL7->3wealth8 | -0.09 | 0.12 | 0.03 | 6.31 | 400.00 | 0.24 | 0.09 | 0.33 | 3.51 | 27.27 | 0.43 | 0.09 | 0.52 | 5.27 | 17.31 |
| HL7->4wealth8 | 0.07 | 0.16 | 0.23 | 7.14 | 69.57 | 0.56 | 0.13 | 0.69 | 4.55 | 18.84 | 0.67 | 0.12 | 0.79 | 5.85 | 15.19 |
| HL7->5wealth8 | -0.12 | 0.22 | 0.10 | 7.70 | 220.00 | 0.44 | 0.21 | 0.65 | 5.17 | 32.31 | 0.37 | 0.16 | 0.53 | 5.99 | 30.19 |
| **SEP at Wave 8 as mediated by the quality of life (hearing aids some of the time 17**.**15%, n=566)** | | | | | | **(hearing aids use some of the time 23**.**17%, n=457)** | | | | | **(hearing aids use some of the time 22**.**90%, n=481)** | | | | |
| HL7->1wealth8 | (Reference) | | | | |  |  |  |  |  |  |  |  |  |  |
| HL7->2wealth8 | 0.02 | 0.09 | 0.11 | 1.90 | 81.82 | 0.19 | 0.05 | 0.24 | 0.94 | 20.83 | 0.37 | 0.04 | 0.41 | 1.04 | 9.76 |
| HL7->3wealth8 | 0.06 | 0.08 | 0.14 | 1.45 | 57.14 | 0.31 | 0.04 | 0.35 | 0.77 | 11.43 | 0.56 | 0.02 | 0.58 | 0.45 | 3.45 |
| HL7->4wealth8 | 0.20 | 0.11 | 0.31 | 2.30 | 35.48 | 0.64 | 0.07 | 0.71 | 1.30 | 9.86 | 0.85 | 0.04 | 0.89 | 1.04 | 4.49 |
| HL7->5wealth8 | 0.10 | 0.04 | 0.14 | -0.70 | 28.57 | 0.59 | 0.08 | 0.67 | -1.16 | 11.94 | 0.61 | 0.08 | 0.69 | -1.52 | 11.59 |
| **SEP at Wave 8 as mediated by the quality of life (hearing aids use most of the time 10%, n=330)** | | | | | | **(hearing aids use most of the time 13**.**69%, n=270)** | | | | | **(hearing aids use most of the time 13**.**19%, n=277)** | | | | |
| HL7->1wealth8 | (Reference) | | | | |  |  |  |  |  |  |  |  |  |  |
| HL7->2wealth8 | 0.04 | 0.02 | 0.06 | 0.47 | 33.33 | 0.21 | 0.00 | 0.21 | 0.00 | 0.00 | 0.46 | 0.06 | 0.52 | -1.60 | 11.54 |
| HL7->3wealth8 | 0.06 | 0.05 | 0.11 | 1.38 | 45.45 | 0.30 | 0.04 | 0.34 | 0.87 | 11.76 | 0.62 | 0.05 | 0.67 | -1.16 | 7.46 |
| HL7->4wealth8 | 0.17 | 0.12 | 0.29 | 3.07 | 41.38 | 0.63 | 0.08 | 0.71 | 1.70 | 11.27 | 0.90 | 0.02 | 0.92 | -0.62 | 2.17 |
| HL7->5wealth8 | 0.08 | 0.03 | 0.11 | 0.59 | 27.27 | 0.58 | 0.03 | 0.61 | -0.62 | 4.92 | 0.64 | 0.06 | 0.70 | -1.44 | 8.57 |
| **DEPR at Wave 8 due to HL, according to wealth (quintiles)** | | | | | |  |  |  |  |  |  |  |  |  |  |
| HL7->DEPR8 | 0.15 | | | | | 0.01 | | | | | 0.16 | | | | |
| QoL7(HL8)->DEPR8 for 1wealth8 |  | (Reference) | | | |  | (Reference) | | | |  | (Reference) | | | |
| QoL7(HL8)->DEPR8 for 2wealth8 | 0.51  (direct effect) | 0.01 | 0.52 | 0.65 | 1.92 | 0.50  (direct effect) | 0.01 | 0.51 | 0.44 | 1.96 | 0.50  (direct effect) | 0.01 | 0.51 | 0.50 | 1.96 |
| QoL7(HL8)->DEPR8 for 3wealth8 |  | 0.14 | 0.65 | 3.23 | 21.54 |  | 0.14 | 0.64 | 2.37 | 21.88 |  | 0.11 | 0.61 | 2.49 | 18.03 |
| QoL7(HL8)->DEPR8 for 4wealth8 |  | 0.18 | 0.69 | 3.02 | 26.09 |  | 0.23 | 0.73 | 2.84 | 31.51 |  | 0.14 | 0.64 | 2.26 | 21.88 |
| QoL7(HL8)->DEPR8 for 5wealth8 |  | 0.38 | 0.89 | 4.24 | 42.70 |  | 0.48 | 0.98 | 3.37 | 48.98 |  | 0.36 | 0.86 | 4.19 | 41.86 |
| (Continued) | | | | | | | | | | | | | | | |
|  | | | | | | | | | | | | | | | |
| **DEPR at Wave 8 due to HL, according to wealth in quintiles (hearing aids some of the time 17**.**15%, n=566)** | | | | | | **(hearing aids use some of the time 23**.**17%, n=457)** | | | | | **(hearing aids use some of the time 22**.**90%, n=481)** | | | | |
|  | **Self-reported HL*** | | | | | **Self-reported HL (improved measures**)** | | | | | **Objective HL (via HearCheck Screener)***** | | | | |
| QoL7(HL8)->DEPR8 for 1wealth8 |  | (Reference) | | | |  | (Reference) | | | |  | (Reference) | | | |
| QoL7(HL8)->DEPR8 for 2wealth8 | 0.51  (direct effect) | 0.03 | 0.54 | 0.74 | 5.56 | 0.50  (direct effect) | 0.03 | 0.53 | 0.65 | 5.66 | 0.50  (direct effect) | 0.01 | 0.51 | 0.45 | 1.96 |
| QoL7(HL8)->DEPR8 for 3wealth8 |  | 0.09 | 0.60 | 1.35 | 15.00 |  | 0.07 | 0.57 | 0.75 | 12.28 |  | 0.02 | 0.52 | 0.44 | 3.85 |
| QoL7(HL8)->DEPR8 for 4wealth8 |  | 0.12 | 0.63 | 1.40 | 19.05 |  | 0.12 | 0.62 | 1.23 | 19.35 |  | 0.05 | 0.55 | 0.96 | 9.09 |
| QoL7(HL8)->DEPR8 for 5wealth8 |  | 0.08 | 0.59 | -0.70 | 13.56 |  | 0.19 | 0.69 | -0.77 | 27.54 |  | 0.17 | 0.67 | -0.82 | 25.37 |
| **DEPR at Wave 8 due to HL, according to wealth in quintiles (hearing aids use most of the time 10%, n=330)** | | | | | | **(hearing aids use most of the time 13**.**69%, n=270)** | | | | | **(hearing aids use most of the time 13**.**19%, n=277)** | | | | |
|  |  | | | | |  | | | | |  | | | | |
| QoL7(HL8)->DEPR8 for 1wealth8 |  | (Reference) | | | |  | (Reference) | | | |  | (Reference) | | | |
| QoL7(HL8)->DEPR8 for 2wealth8 | 0.51  (direct effect) | 0.01 | 0.52 | 0.42 | 1.92 | 0.50  (direct effect) | 0.00 | 0.50 | 0.00 | 0.00 | 0.50  (direct effect) | 0.01 | 0.51 | -0.48 | 1.96 |
| QoL7(HL8)->DEPR8 for 3wealth8 |  | 0.07 | 0.58 | 1.06 | 12.07 |  | 0.06 | 0.56 | 0.84 | 10.71 |  | 0.05 | 0.55 | -1.07 | 9.09 |
| QoL7(HL8)->DEPR8 for 4wealth8 |  | 0.13 | 0.65 | 2.26 | 20.00 |  | 0.13 | 0.63 | 1.54 | 20.63 |  | 0.02 | 0.52 | -0.60 | 3.85 |
| QoL7(HL8)->DEPR8 for 5wealth8 |  | 0.05 | 0.56 | 0.58 | 8.93 |  | 0.08 | 0.58 | -0.61 | 13.79 |  | 0.12 | 0.62 | -1.32 | 1..35 |
| *Current categories of self-reported data: the sum of those that rated their hearing as fair or poor on a five-point Likert scale (excellent, very good, good, fair or poor), or responded positively in the question whether they find it difficult to follow a conversation if there is background noise (such as TV, radio or children playing).  **Improved categorisation of self-reported data: the sum of those that rated their hearing as fair or poor on a five-point Likert scale (excellent, very good, good, fair or poor), or responded that they have moderate or great difficulty in following a conversation if there is background noise (such as TV, radio or children playing).  ***Objective HL: >35dB HL at 3.0 kHz, in the better-hearing ear.  **QoL**: CASP-19 confirmatory factor analyses factor score in Wave 7; functioned as an endogenous mediator variable that intervenes between HL and wealth, explaining the relation between HL and SEP  **Wealth8**: socioeconomic position according to wealth in Wave 8; functioned both as an endogenous outcome variable and a moderating/intermediate dependent variable of depression (1 represents the highest quintile; 5 represents the lowest quintile).  **DEPR8**: exogenous outcome variable; represents participants with CES-D Score ⩾4 in Wave 2. | | | | | | | | | | | | | | | |

**Figure 1**


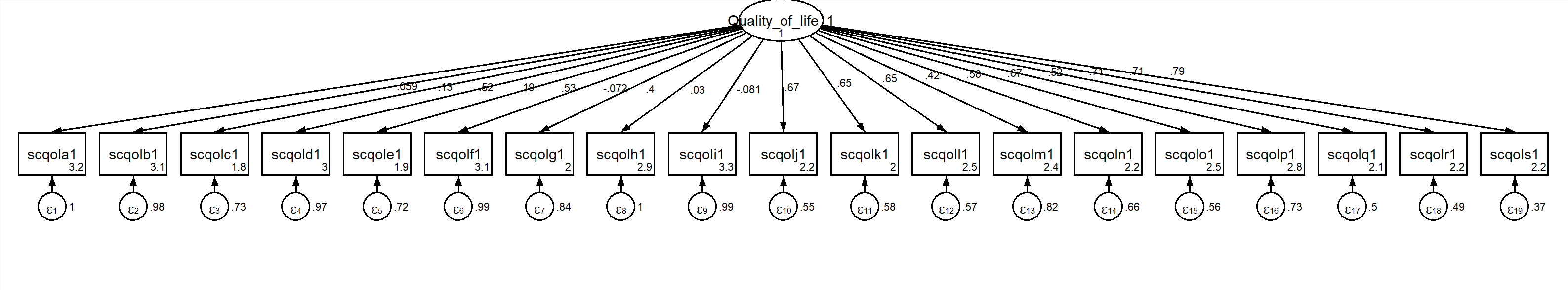


**Figure 2**


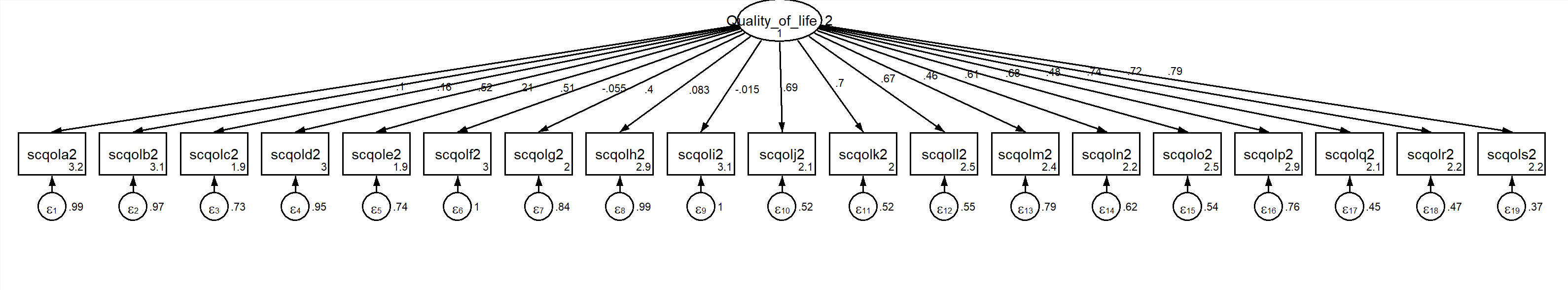


**Figure 3**


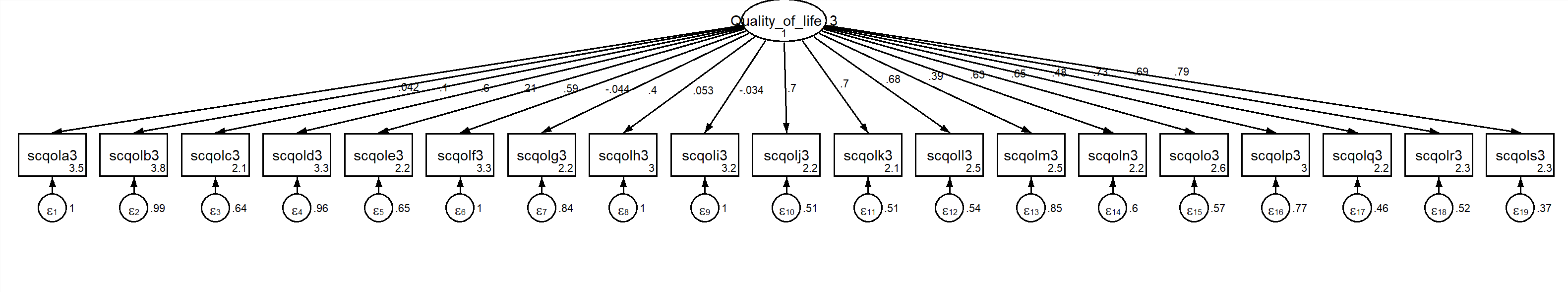


**Figure 4**


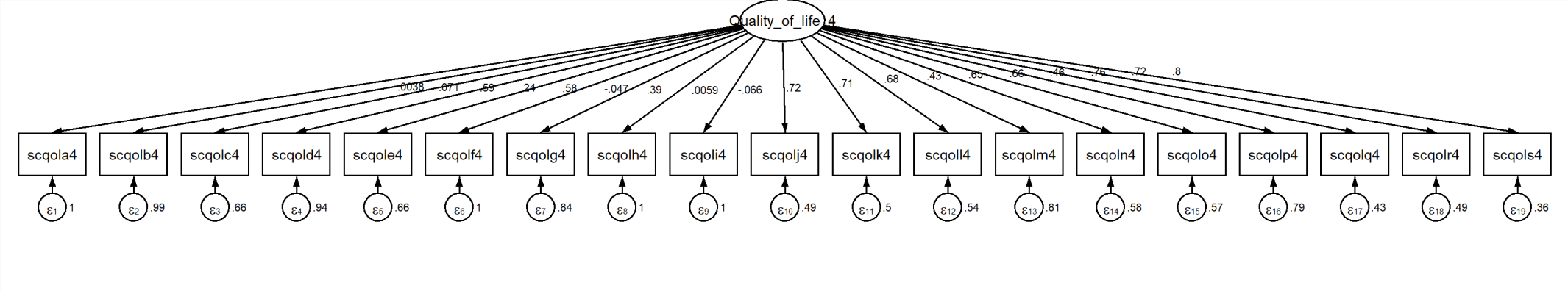


**Figure 5**


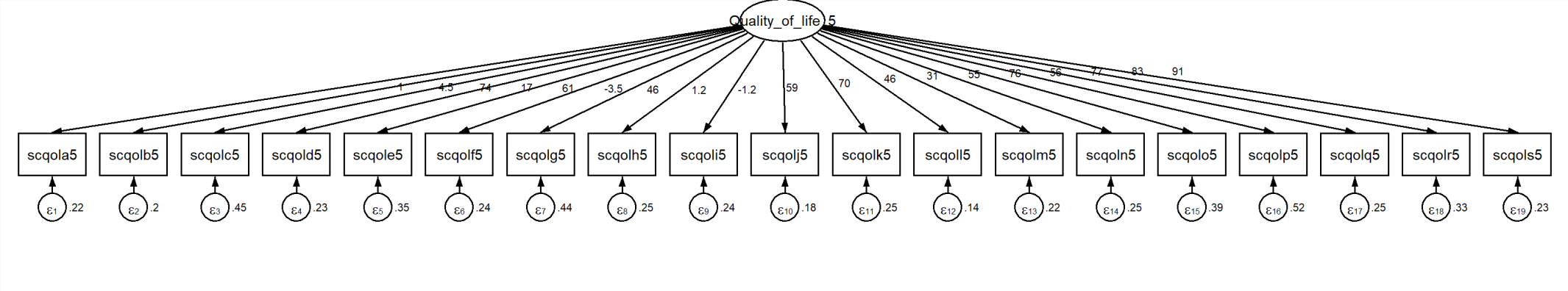


**Figure 6**


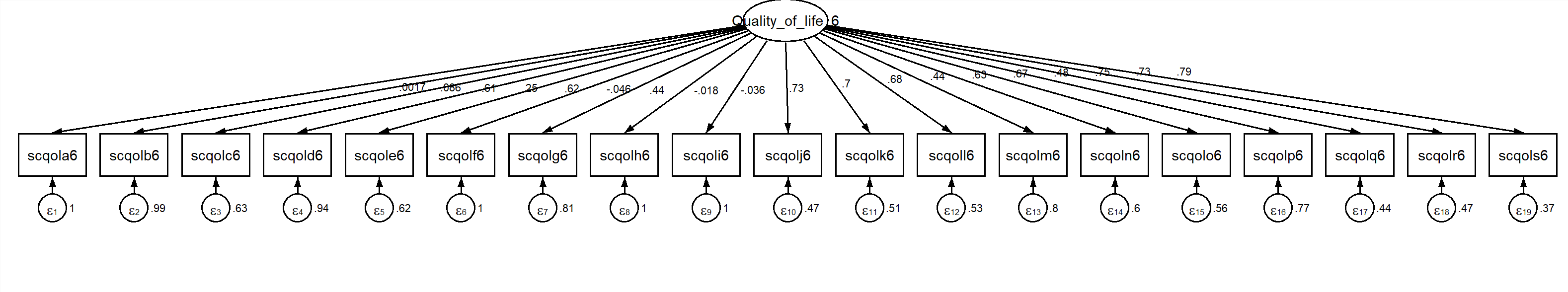


**Figure 7**


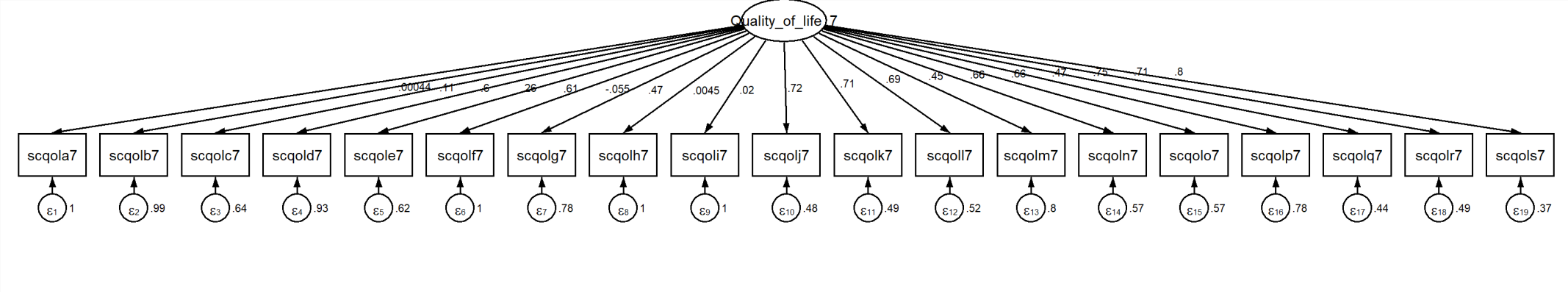


**Figure 8**


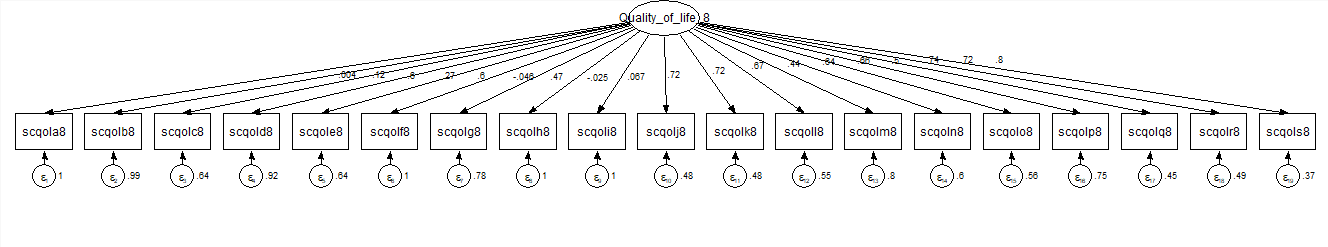


**Figures 1-8**. Factor scoring coefficients of Quality of Life latent variables (standardized beta weights of the confirmatory factor analysis for the 19-items of CASP-19) in the 8 waves of English Longitudinal Study of Ageing (ELSA)

**Figure 9**


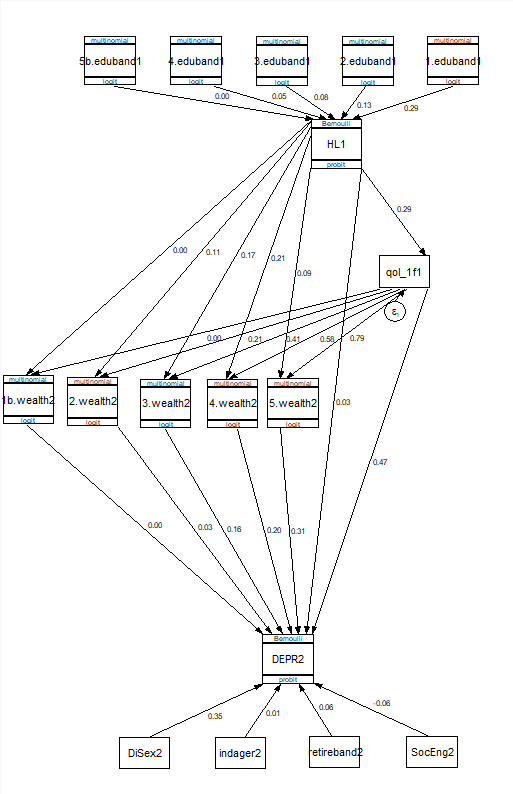


**Figure 10**


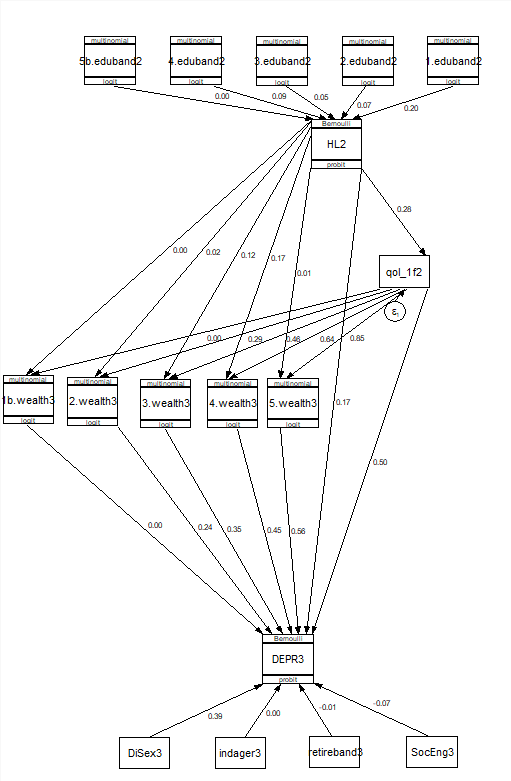


**Figure 11**


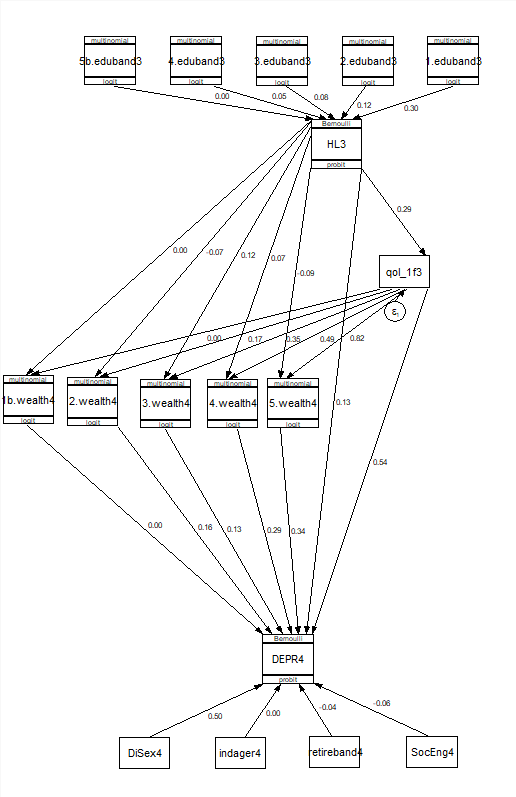


**Figure 12**


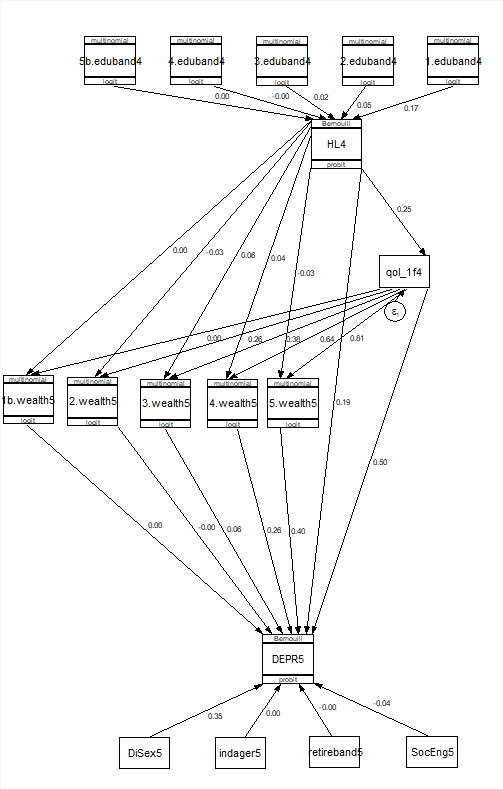


**Figure 13**


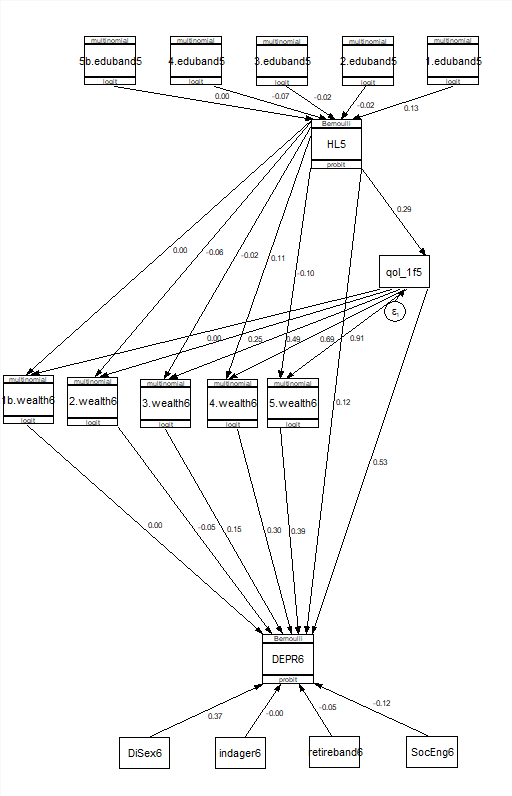


**Figure 14**


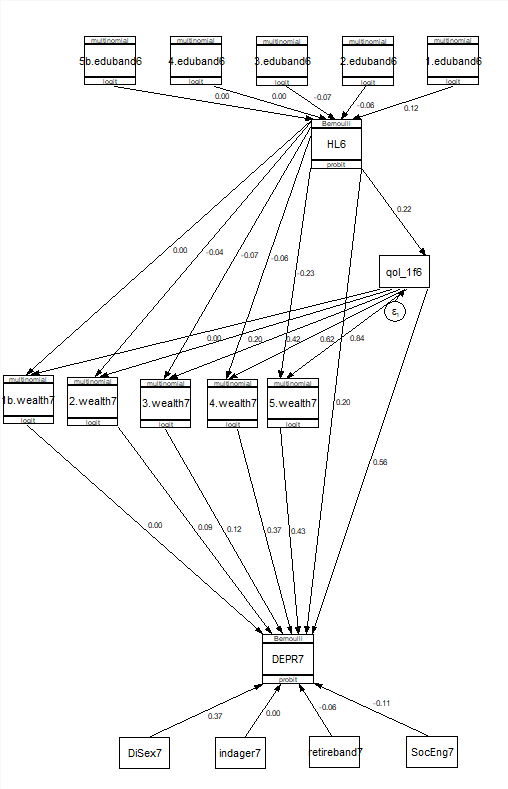


**Figure 15**


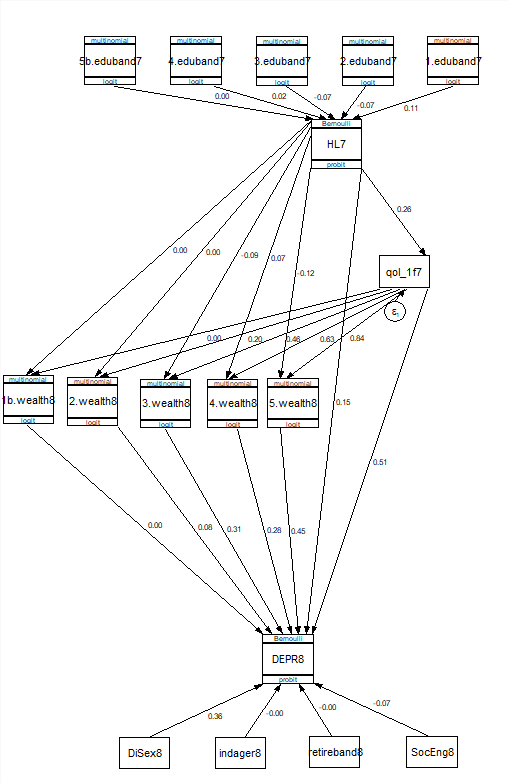


**Figures 9-15**. Factor scoring coefficients (Standardized beta weights) of the generalised structural equation model representing the dynamic relationship between hearing loss, quality of life, socioeconomic position and depression in 8 Waves of English Longitudinal Study of Ageing (ELSA)

**Figure 16**


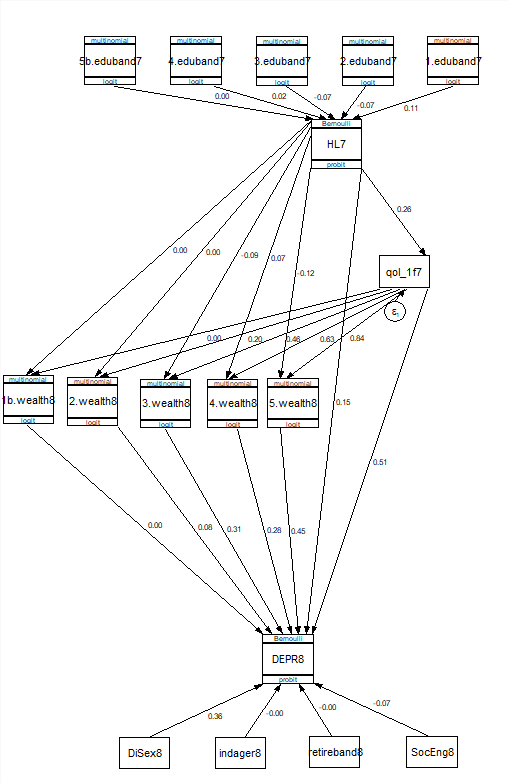

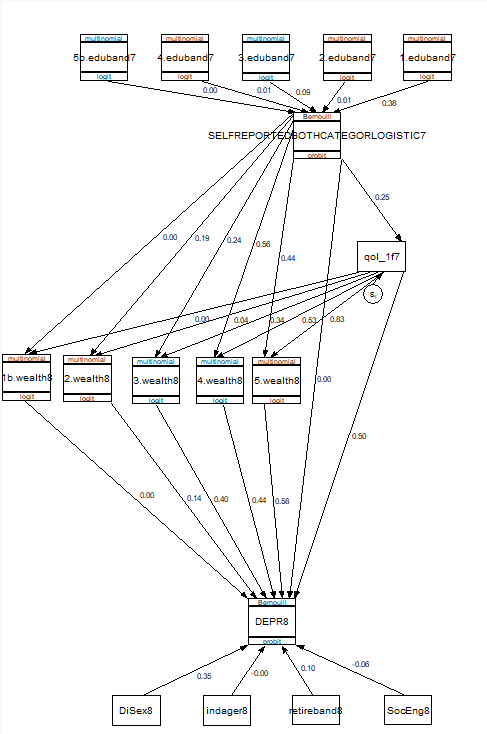

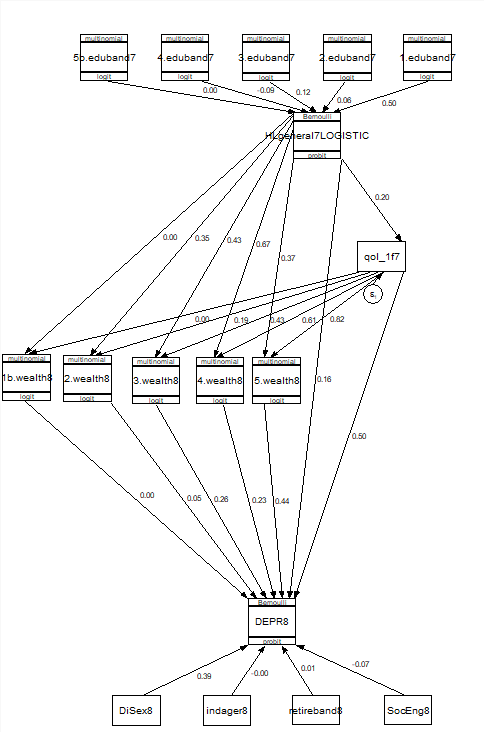


**Figure 16** Factor scoring coefficients (Standardized beta weights) of the structural equation model representing the dynamic relationship between hearing loss, quality of life, socioeconomic position and depression according to different HL measures in 7th Wave of English Longitudinal Study of Ageing (ELSA)

**Figure 17**


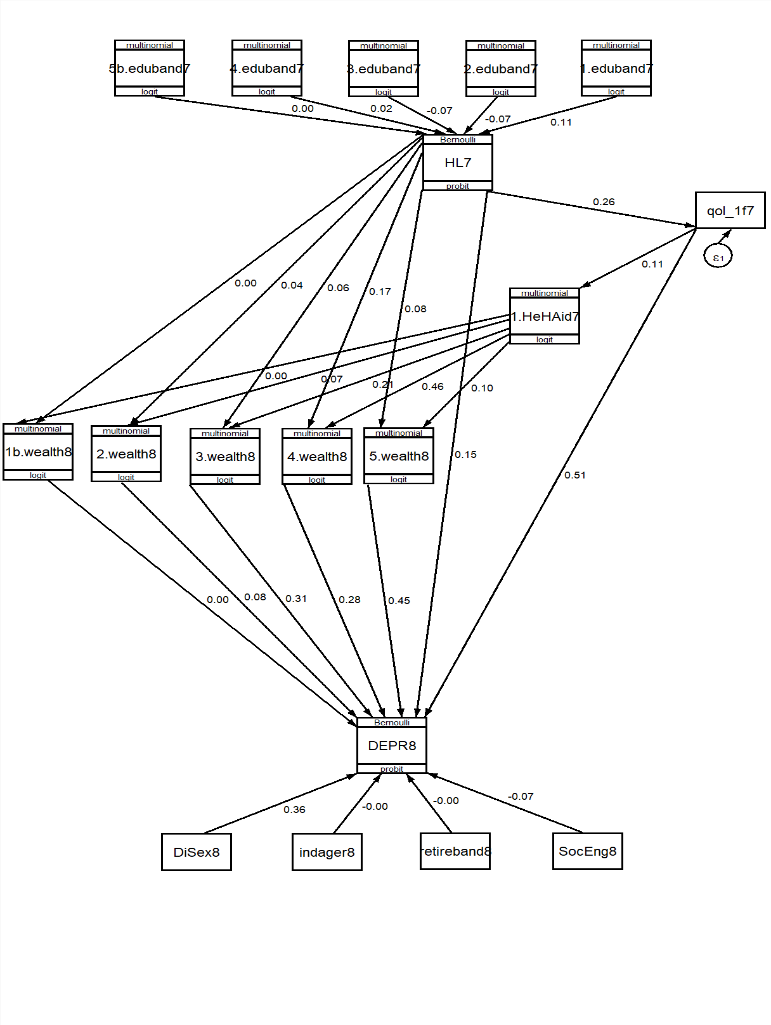

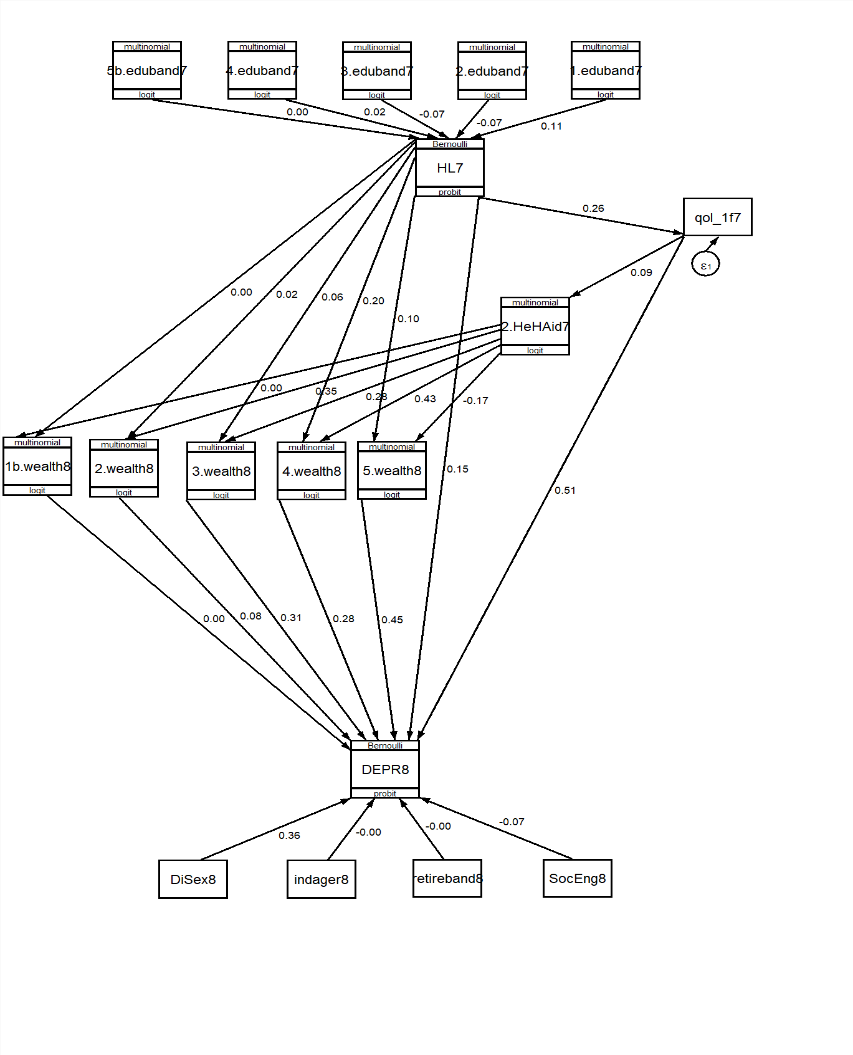


**Figure 17** Factor scoring coefficients (Standardized beta weights) of the structural equation model representing the dynamic relationship between **self-reported hearing loss**, quality of life, socioeconomic position and depression, moderated by hearing aid use (a. most of the time, b. some of the time) in the 7th Wave of English Longitudinal Study of Ageing (ELSA)

**Figure 18**


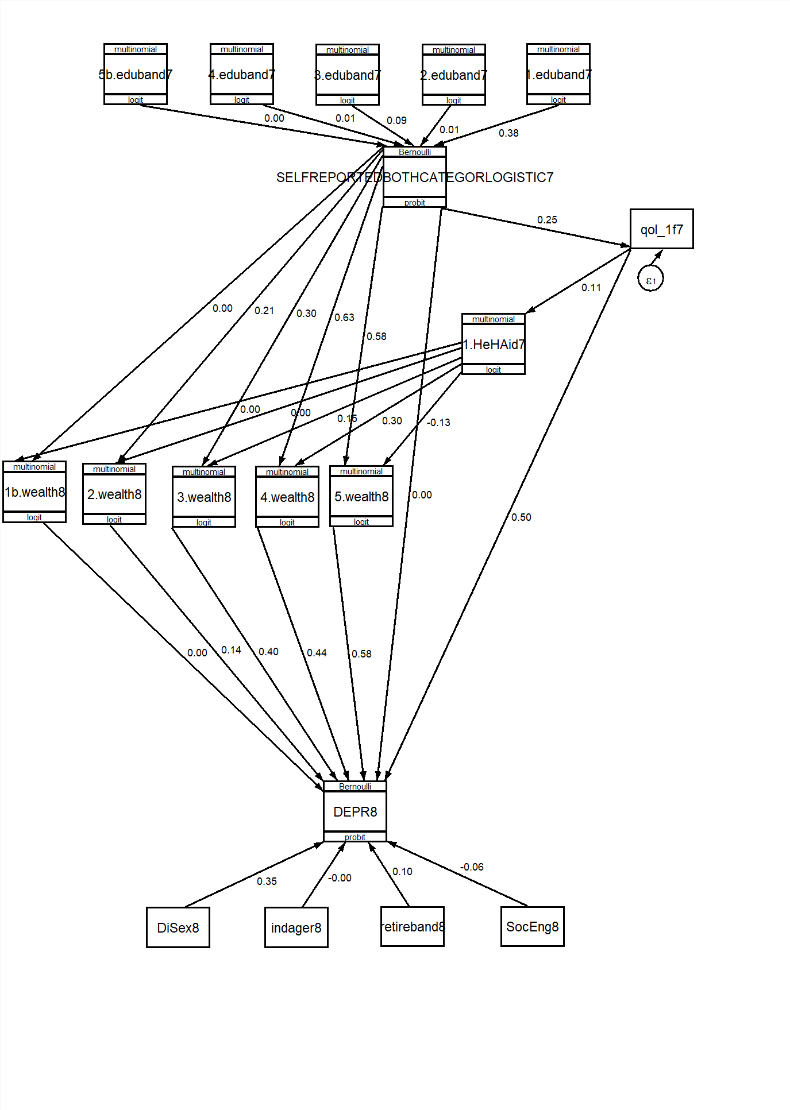

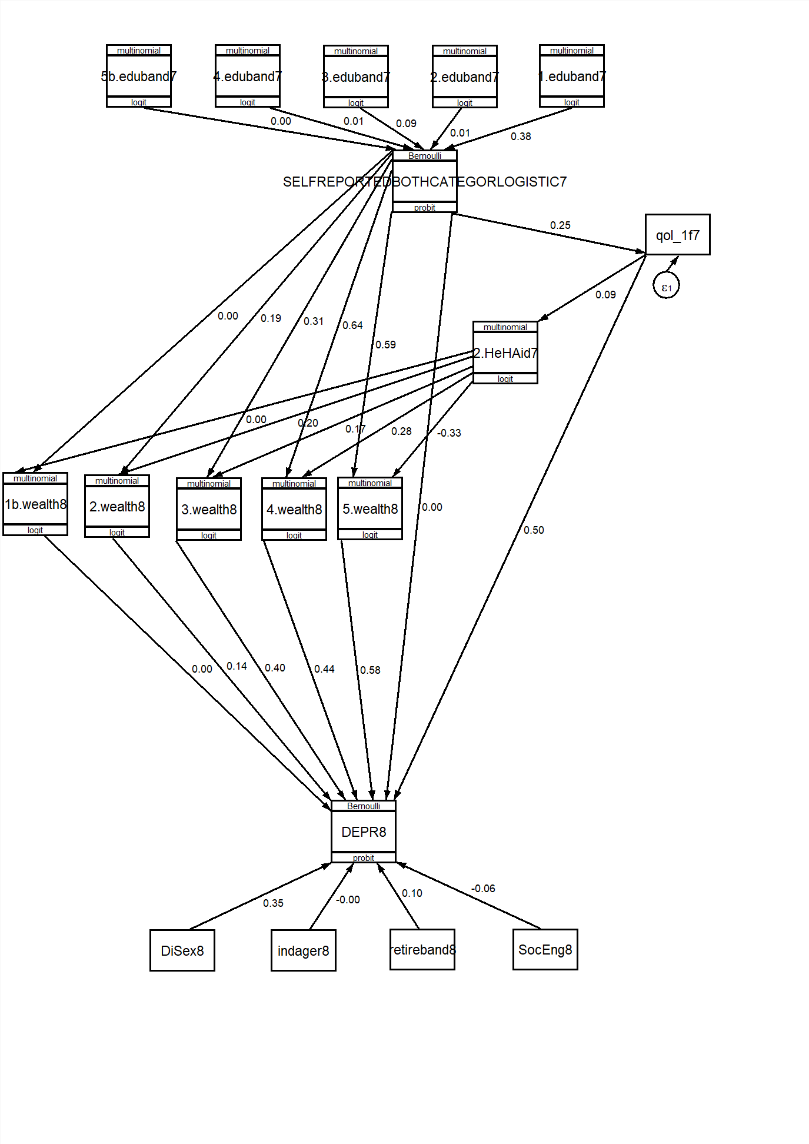


**Figure 18** Factor scoring coefficients (Standardized beta weights) of the structural equation model representing the dynamic relationship between **improved self-reported hearing loss**, quality of life, socioeconomic position and depression, moderated by hearing aid use (a. most of the time, b. some of the time) in the 7th Wave of English Longitudinal Study of Ageing (ELSA)

**Figure 19**


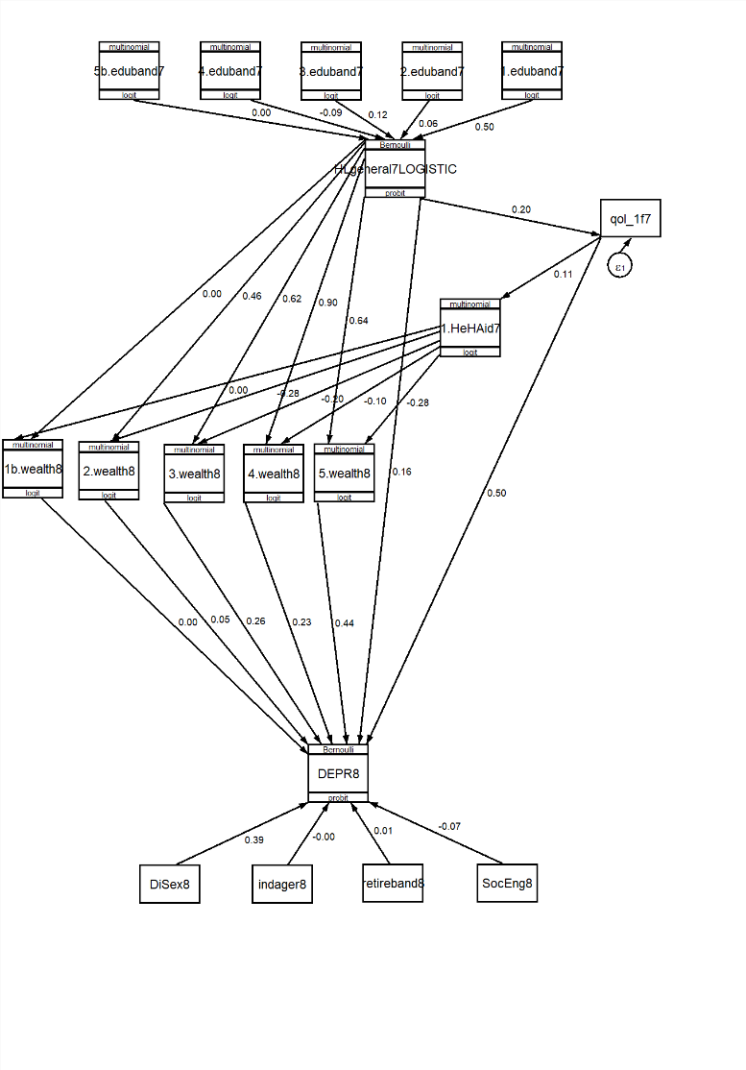

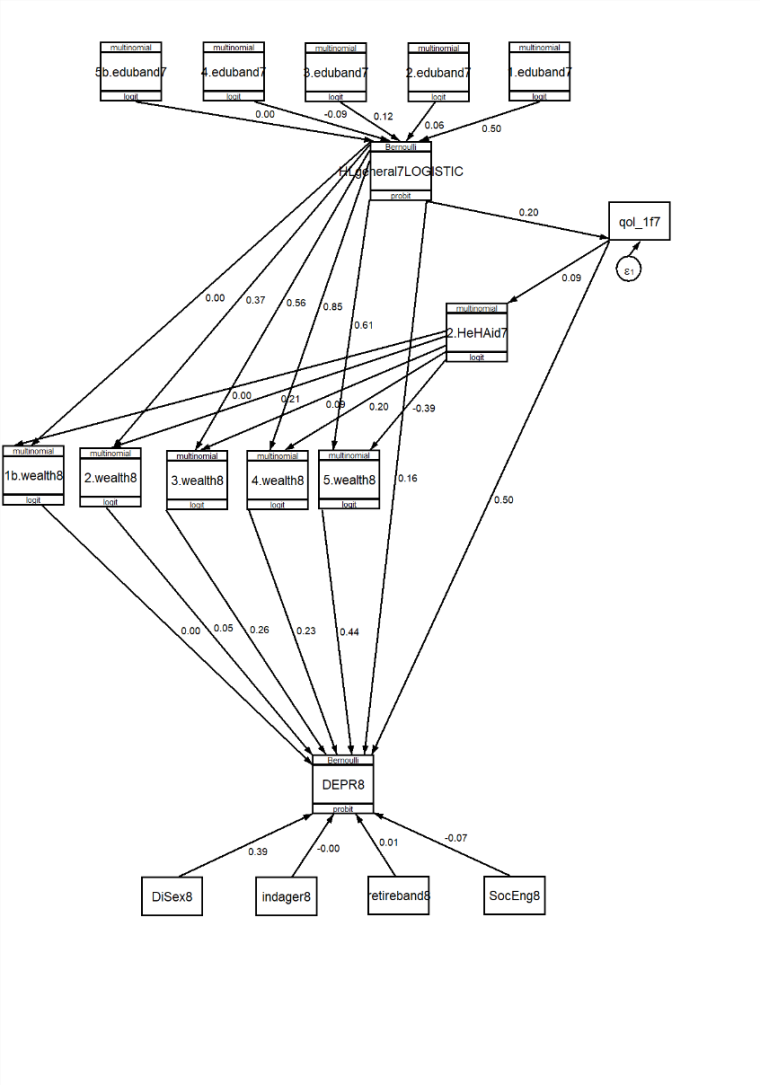


**Figure 19** Factor scoring coefficients (Standardized beta weights) of the structural equation model representing the dynamic relationship between **objectively measured hearing loss**, quality of life, socioeconomic position and depression, moderated by hearing aid use (a. most of the time, b. some of the time) in the 7th Wave of English Longitudinal Study of Ageing (ELSA)

1. Coefficient (b): Weak = 0.00 to 0.29, Low = 0.30 to 0.49, Moderate = 0.50 to 0.69, Strong = 0.70 to 0.89, Very Strong = 0.90 to 1.00 (Pett, 2015) [↑](#endnote-ref-1)
2. Sobel test larger than 1.96 in absolute value is significant at the .05 level [↑](#endnote-ref-2)
3. The percentage of the total effect that is mediated (indirect effect/ total effect) [↑](#endnote-ref-3)
4. All models were adjusted for age, gender, retirement status and social engagement [↑](#endnote-ref-4)
5. QoL: Quality of life (CASP-19 confirmatory factor analyses factor score) [↑](#endnote-ref-5)
6. HL: Hearing loss [↑](#endnote-ref-6)
7. Numbers *after* each variable indicate the Wave in ELSA [↑](#endnote-ref-7)
8. SEP: categories of wealth (1st quintile highest; 5th quintile lowest) [↑](#endnote-ref-8)
9. Wealth: represents the value of the primary house minus the outstanding primary house mortgage, the value of savings and shares minus depts, and the value of other properties and businesses, also known as the sum of net financial, physical and housing wealth [↑](#endnote-ref-9)
10. DEPR: CES-D Score ⩾4 [↑](#endnote-ref-10)
11. Coefficient (b): Weak = 0.00 to 0.29, Low = 0.30 to 0.49, Moderate = 0.50 to 0.69, Strong = 0.70 to 0.89, Very Strong = 0.90 to 1.00 (Pett, 2015) [↑](#endnote-ref-11)
12. Sobel test larger than 1.96 in absolute value is significant at the .05 level [↑](#endnote-ref-12)
13. The percentage of the total effect that is mediated (indirect effect/ total effect) [↑](#endnote-ref-13)
